# Narrowband microwave-photonic notch filters using Brillouin-based signal transduction in silicon

**DOI:** 10.1038/s41467-022-29590-0

**Published:** 2022-04-11

**Authors:** Shai Gertler, Nils T. Otterstrom, Michael Gehl, Andrew L. Starbuck, Christina M. Dallo, Andrew T. Pomerene, Douglas C. Trotter, Anthony L. Lentine, Peter T. Rakich

**Affiliations:** 1 Department of Applied Physics, Yale University, New Haven, CT 06520 USA; 2 Photonic and Phononic Microsystems, Sandia National Laboratories, Albuquerque, NM 87185 USA

**Keywords:** Integrated optics, Microwave photonics, Nonlinear optics

## Abstract

The growing demand for bandwidth makes photonic systems a leading candidate for future telecommunication and radar technologies. Integrated photonic systems offer ultra-wideband performance within a small footprint, which can naturally interface with fiber-optic networks for signal transmission. However, it remains challenging to realize narrowband (∼MHz) filters needed for high-performance communications systems using integrated photonics. In this paper, we demonstrate all-silicon microwave-photonic notch filters with 50× higher spectral resolution than previously realized in silicon photonics. This enhanced performance is achieved by utilizing optomechanical interactions to access long-lived phonons, greatly extending available coherence times in silicon. We use a multi-port Brillouin-based optomechanical system to demonstrate ultra-narrowband (2.7 MHz) notch filters with high rejection (57 dB) and frequency tunability over a wide spectral band (6 GHz) within a microwave-photonic link. We accomplish this with an all-silicon waveguide system, using CMOS-compatible fabrication techniques.

## Abstract (web summary)

It remains challenging to realize narrowband filters needed for high-performance communications systems using integrated photonics. Using a multi-port Brillouin-based optomechanical system, the authors demonstrate an ultra-narrowband notch filter with high rejection with CMOS compatible techniques.

## Introduction

Microwave-photonic systems offer a range of capabilities that are difficult to achieve using conventional radio frequency (RF) signal-processing technologies. Low-loss photonic systems can enable the transmission of wideband signals over large distances with low power consumption, and immunity to RF interference^1–4^. If these advantages can be realized using integrated photonics, complex microwave-photonic systems could be implemented within a small footprint by leveraging well-established manufacturing techniques in the rapidly growing field of integrated photonics^5^. However, there remains a need for integrated-photonic technologies that can produce narrowband filtering operations^6^. Such narrowband filters play an important role in modern communications and radar systems. For example, narrowband notch filters are used to suppress unwanted interfering signals that can degrade (or overwhelm) a communications system^7^. It is challenging to perform narrowband (~MHz) signal-processing operations using integrated photonic circuits because exceedingly low optical losses (0.001 dB/cm) are needed to store optical photons at microsecond timescales. To date, all-optical microwave-photonic notch filters implemented using silicon and silicon nitride waveguide resonators have produced linewidths ≥150 MHz^8,9^.

An alternative approach to achieving narrowband filtering in an integrated-photonic platform is by utilizing stimulated Brillouin scattering (SBS), namely the optomechanical coupling of light and traveling acoustic waves^10–12^. The long lifetime of the acoustic modes participating in the interaction produces narrow spectral features, which can be used to produce microwave-photonic filters, analogous to the role played by surface acoustic wave (SAW) and bulk acoustic wave (BAW) devices in microwave signal processing^13,14^. Specifically, Brillouin-based notch filtering has been demonstrated by using narrowband Brillouin-induced loss or gain to manipulate the microwave sidebands encoded on an optical carrier. While impressive filtering schemes have been demonstrated in chalcogenide-waveguide systems using backward Brillouin interactions^15–20^, such microwave-photonic systems face significant barriers to full integration. Chalcogenide materials suffer from many unwanted effects (such as photodarkening and photosensitivity) as well as poor aging characteristics^21,22^, and these materials are not found in integrated-photonic foundries. Furthermore, such schemes require isolators, circulators, advanced modulation schemes, and narrowband optical filters with high power handling^16,17,23,24^, which are often difficult to realize in integrated photonics. To make narrowband microwave-photonic filters a feasible and scalable technology, it is necessary to implement the photonic circuits in a reliable foundry-compatible platform such as silicon photonics.

In this work, we show that many of these issues can be addressed by using a new signal processing scheme that utilizes Brillouin interactions within a multi-port photonic-phononic emitter-receiver (PPER) device^25^. The PPER-based scheme offers new design degrees of freedom relative to prior Brillouin-based microwave-photonic filters^26^, producing narrowband, tunable, and tailorable notch filters. In a PPER device, forward Brillouin scattering is used to transduce information between two spatially separated waveguides, producing a narrowband frequency response set by the properties of the acoustic mode^25,27^. By incorporating the PPER within an interferometer, we achieve signal cancellation within a narrow spectral range, enabling notch filters with bandwidths as narrow as 2.7 MHz and signal rejection as high as 57 dB.

The forward Brillouin geometry used here—in which all optical fields are co-propagating—does not require circulators^28^, and the unique properties of the PPER device obviate the requirement of pump-rejection filtering^26^. Additionally, we show how the notch frequency can be shifted over multiple GHz by using an optical local oscillator at the filter input, without degrading the narrowband (~MHz) filter resolution. Furthermore, we demonstrate how acoustic mode engineering enables tailoring of the frequency response of the filter, achieving a flat pass-band, and enables the design of multi-pole filters. We characterize a full microwave-photonic link implementing a PPER-based filter, demonstrating a link gain of −3.6 dB, and study the link noise figure and dynamic range, showing the feasibility of these filters to be integrated within larger microwave-photonic systems. The devices used in this work were fabricated in a standard silicon-on-insulator (SOI) platform, using well-established fabrication methods and waveguide geometries, opening the door to wafer-scale integration of additional system components on the same platform.

## Results

### Operation scheme

**Fig. 1:**
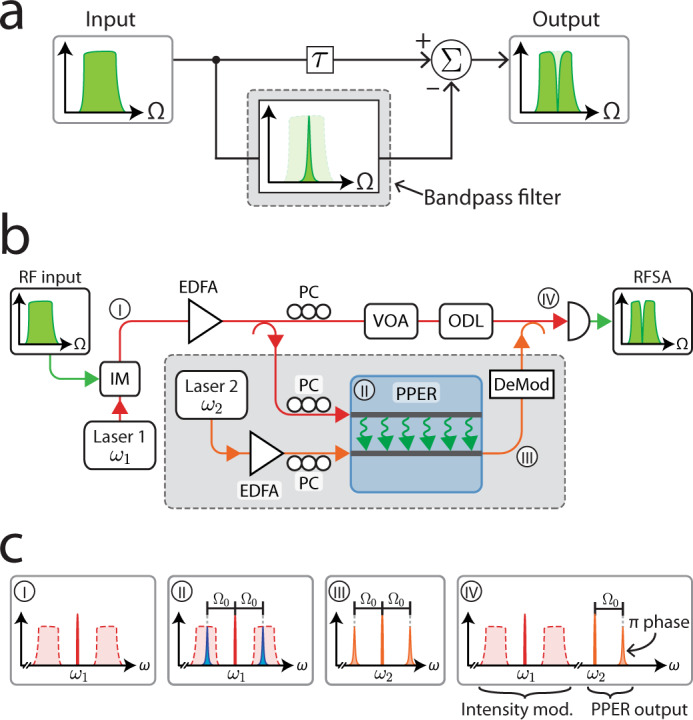
Operation scheme of a PPER-based microwave-photonic notch-filter. **a** Operation scheme of the notch filter. The signal is split into two paths, one of which is bandpass filtered. By combining the two signals out of phase, a notch filter frequency response is obtained. **b** Schematic illustration of the experimental setup used to demonstrate PPER-based notch filtering. The input RF tone is modulated onto an optical carrier using an intensity modulator (IM). The optical signal is split into two paths: One path is bandpass filtered using a PPER-based filter (shaded region), and the second path is controlled using an optical delay line (ODL) to achieve signal cancellation at the detector. VOA: variable optical attenuator, EDFA: erbium-doped fiber amplifier, PC: polarization controller, DeMod: phase demodulation (bandpass filtering), RFSA: RF spectrum analyzer. **c** Optical spectrum at different stages of the signal path: (I) Wideband RF signal modulated on an optical carrier. (II) The acoustic response (blue) transduces a narrow spectral band. (III) The resulting narrowband phase modulation. (IV) At the detector, the wideband intensity-modulated signal (red) is combined with the bandpass-filtered signal (orange).

The essential operating principle of the Brillouin-based notch filter relies on interferometric cancellation, as illustrated in Fig. 1a. Through this scheme, an incident wideband signal is split into two paths: one arm of the interferometer contains a bandpass filter that only transmits portions of the signal at the desired notch filter frequency, while the other arm of the interferometer transmits the entire wideband signal. The two arms of the interferometer are combined at the output, setting their relative signal delay to obtain complete cancellation of the portion of the spectrum that was transmitted through the bandpass filter. In this way, the frequency response of the bandpass filter is effectively inverted to achieve the desired notch filter response.

Here, we implement such interferometric cancellation using a silicon PPER device, as seen in the experimental scheme illustrated in Fig. 1b. The input RF signal is modulated onto an optical carrier which is split into two optical paths: one path (lower arm in Fig. 1) is bandpass filtered using the PPER device—resulting in a frequency response of a few MHz^11,29^—while the second path propagates without filtering (upper arm in Fig. 1). The relative time delay between the signals in the two interferometer arms is controlled using an ODL to produce signal cancellation. The two optical waves are combined and directed to a photodetector, resulting in an output RF signal following a notch frequency response, with a bandwidth set by the PPER filter.

More specifically, the input RF signal is encoded onto an optical carrier with frequency *ω*_1_ using an intensity modulator (Fig. 1c, panel (I)), which is then split into two separate optical paths. The optical wave in one of the paths is injected into the ‘emit’ waveguide of the PPER. Within the PPER device, the intensity-modulated light drives a narrowband coherent acoustic mode through forward Brillouin scattering (Fig. 1c, panel (II)). A separate laser source with frequency *ω*_2_ is injected into the ‘receive’ waveguide of the PPER, and the light propagating in the device is phase-modulated by the driven acoustic wave, resulting in narrow optical sidebands (Fig. 1c, panel (III)). The frequency response of the PPER device is determined by the properties of the acoustic mode taking part in the Brillouin scattering process, namely its resonant frequency (Ω_0_) and dissipation rate. The phase-modulated light exiting the ‘receive’ waveguide is demodulated using an optical bandpass filter (by suppressing one of the optical phase-modulation sidebands). The light from the two optical paths is combined (Fig. 1c, panel (IV)) and directed to a photodetector.

The light from each of the optical paths results in an RF signal at the detector; a wideband signal from the non-filtered light at frequency *ω*_1_, and a narrowband signal around the Brillouin frequency (Ω_0_) as a result of the demodulated output from the PPER filter at frequency *ω*_2_. By balancing the power and phase of the two signals, the output RF signal is suppressed at the Brillouin frequency, yielding a narrowband notch filter response. We note, that the signal interference occurs in the RF domain (at the detector) and is not the result of optical interference, as the two laser sources are well separated in wavelength (∣*ω*_1_ − *ω*_2_∣ ≫ Ω_0_), such that their beat note is well beyond the detector bandwidth.

In the proof-of-concept demonstrations presented in this work, all measurements were conducted at telecom wavelengths, and light was coupled on and off the chip using integrated grating couplers. Additionally, the interferometer was balanced using a variable optical delay line (ODL) and a variable optical attenuator (VOA); no active stabilization was used. It is important to note that many of these components would not be required when fully integrating the filter on-chip (see “Discussion” for details).

### Filtering demonstration

**Fig. 2:**
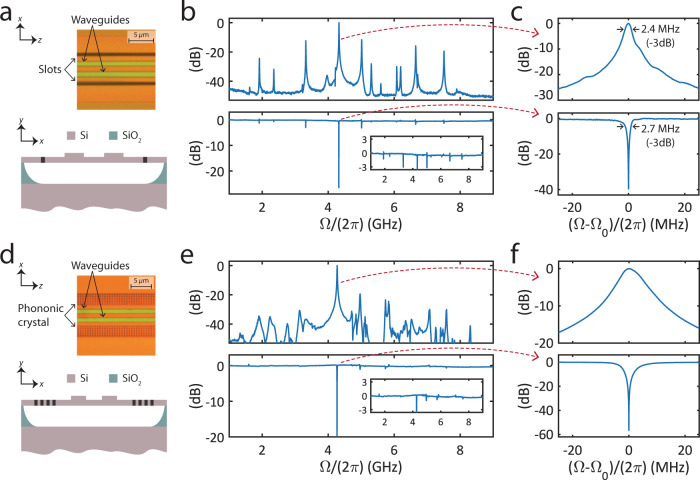
Microwave-photonic frequency response. **a** Top: Top-down micrograph of a PPER device, showing the two ridge waveguides, and the slots used to confine the acoustic mode. Bottom: Illustration of the PPER device cross-section. **b** Top: Measured frequency response of the PPER device, showing multiple acoustic modes. Bottom: The corresponding notch filter frequency response. Inset: Magnified view of the pass-band ripple. **c** Magnified view of the frequency response around Ω_0_ = 4.32 GHz. Top: PPER band-pass response. Bottom: Corresponding notch filter response. **d** Top: Top-down micrograph of a PPER device utilizing a phononic crystal structure to confine the acoustic mode. Bottom: Illustration of the device cross-section. **e** Top: Measured frequency response of the PPER device. Bottom: Corresponding notch filter frequency response. Inset: Magnified view of the pass-band ripple. **f** Magnified view of the frequency response around Ω_0_ = 4.3 GHz.

The first PPER device used to demonstrate narrowband notch filtering was fabricated using a standard SOI process and patterned with stepper photolithography at the MESA facilities of Sandia National Laboratories. As shown in Fig. 2a, the active region of the device consists of two ridge-waveguides, which are suspended by removing the oxide under-cladding, with an active length of *L* = 1.48 mm. Each of the rib waveguides supports a TE-like optical mode, while the suspended region supports acoustic modes, which are confined laterally using etched slots in the silicon layer. Confining the acoustic modes to the suspended region of the device results in long phonon lifetimes (i.e., high-*Q* acoustic modes), corresponding to narrow spectral features in the acoustic frequency response. The overlap of the optical and acoustic fields in the device results in strong forward Brillouin coupling (further details can be found in ref. ^11^).

Within the interferometric notch-filtering scheme, the frequency response of the PPER device determines the spectral width of the notch filter. First, we measure the frequency response of the PPER device alone, using the experimental setup illustrated schematically in Fig. 1b without the upper optical path (i.e., setting the VOA to zero transmission). We sweep the frequency of an RF tone at the input to the intensity modulator and measure the output RF power at the same frequency using a synchronized RF spectrum analyzer. The device produces a bandpass response, revealing multiple acoustic resonances (determined by the device geometry^30^), seen in the top panel of Fig. 2b. Focusing on the resonance at frequency Ω_0_/(2*π*) = 4.32 GHz, we see that the acoustic response has a Lorentzian lineshape with a full-width at half-maximum (FWHM) of 2.4 MHz, seen in the top panel of Fig. 2c, corresponding to an acoustic *Q*-factor of *Q* = 1722.

Next, we produce a notch filter response by utilizing the interferometric scheme from Fig. 1. By tuning the optical power and delay (upper arm of the interferometer in Fig. 1b), we achieve signal suppression of 47 dB at the notch frequency (Ω_0_). Outside the filtered notch, the frequency response is flat over a span of 1.7 GHz, as seen in Fig. 2b. The PPER-based filter suppression is on par with state-of-the-art integrated microwave-photonic filtering demonstrations^7^ while standing out for its high spectral resolution (see Supplement, Section 1).

When analyzing a wider frequency range of the notch filter, the effect of spurious acoustic modes can be observed, yielding pass-band ripple on the order of ~3 dB, as seen in the inset of the bottom panel of Fig. 2b. These spectral features are the result of the interference between the unfiltered signal path (upper arm of the interferometer illustrated in Fig. 1b) and the spurious acoustic modes, as can be seen at frequencies 3.3 and 5 GHz. In certain applications, this distortion can hinder filter operation and a ripple-free pass-band is required.

### Ripple suppression using phononic band engineering

Next, we show that we can greatly reduce ripple within the pass-band using phononic band engineering. Using a different PPER device that has phononic crystal regions in place of the slots, we use the frequency-dependent response of the phononic crystal to suppress the unwanted acoustic modes that are the source of pass-band ripple. One such device is shown in Fig. 2d, where the phononic crystal region—a cubic lattice of air holes with a pitch *a* = 0.6 μm and hole diameter *d* = 0.5 μm—result in a phononic stop-band over a ~2 GHz spectral range^29^. The phononic crystal devices were fabricated using standard electron-beam lithography (for further details, see ref. ^29^).

Such devices greatly suppress the spurious acoustic resonances that produce unwanted ripple, as the acoustic mirrors produced by the phononic crystal regions support high-*Q* acoustic resonances only within the phononic stop-band (~3–5 GHz) where the phononic crystal is highly reflective and acts as an acoustic mirror. Acoustic modes with frequencies outside the acoustic stop-band readily propagate through the phononic crystal regions, resulting in low-*Q* modes and weak acousto-optic interaction.

Measurements of PPER devices utilizing phononic crystal structures reveal suppression of the spurious acoustic resonances. The top panel of Fig. 2e shows the measured bandpass frequency response, demonstrating a sharp peak at frequency Ω_0_/(2*π*) = 4.3 GHz (within the acoustic stop-band), with a FWHM of 8.4 MHz. Acoustic modes outside the phononic stop-band are suppressed by at least 20 dB. When used as a notch filter, the distortions in the pass-band are reduced, and the notch filter passband has a ripple of less than 1 dB, as seen in the bottom panel of Fig. 2e. When analyzing the response around the notch frequency, the filter shows signal suppression of 57 dB, as seen in Fig. 2f.

This demonstration shows how acoustic mode engineering is a powerful tool to shape the PPER-based notch filter frequency response. Additionally, acoustic mode engineering enables the design of PPER devices with multi-pole frequency response filters, by utilizing the interference between multiple phonon modes^25,27,29^. Such PPER devices can readily be used to produce multi-pole microwave-photonic notch filters (see Supplement, Section 3).

### Filter tunability

Up to this point, we have demonstrated microwave-photonic systems that produce a static notch filter. However, many applications require a tunable notch filter frequency. Next, we demonstrate how the notch frequency of the filter can be tuned over a large frequency range by using optical frequency mixing. This is achieved by using an optical local oscillator (LO) to tune the input signal with respect to the Brillouin frequency^29^, selecting the spectral band that will be filtered. Other than the LO at the filter input, which enables the selection of the notch frequency, the operation scheme is identical to that of the static filter.

**Fig. 3:**
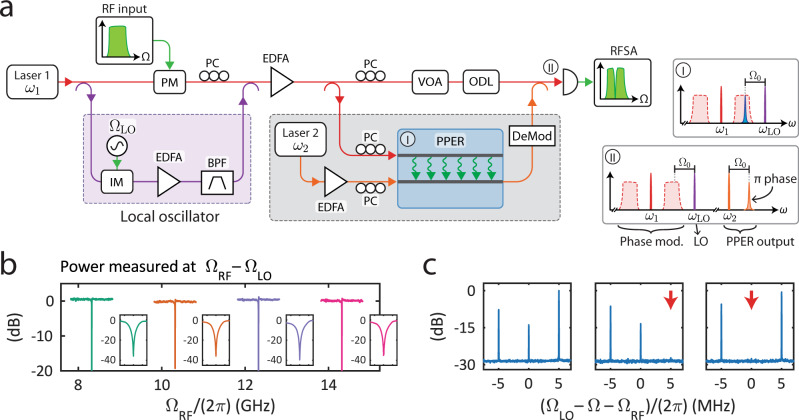
Microwave-photonic notch filter frequency tunability. **a** Schematic illustration of the experimental setup used to tune the notch frequency. The input RF signal is modulated using a phase modulator (PM). An optical local oscillator (LO) tone is synthesized using an intensity modulator (IM) and an optical bandpass filter (BPF). VOA variable optical attenuator, ODL optical delay line, EDFA erbium-doped fiber amplifier, PC polarization controller, DeMod phase demodulation (bandpass filtering), RFSA RF spectrum analyzer. **b** Aggregated data from four measurements demonstrating the tunability of the filter notch frequency over a wide range. For each measurement, the LO was set and the input signal swept a 1 GHz span around the notch frequency. The insets show a magnified view (6 MHz span) of the filter stop-band at each LO setting, demonstrating ∼40 dB extinction. **c** Experimental demonstration of high-resolution filtering. The input RF signal consists of three tones, separated in frequency by 5 MHz (left panel). By tuning the notch filter to the frequency indicated by the red arrow (middle and right panels), one of the tones can be suppressed without affecting the other tones.

The experimental scheme used for the frequency tuning demonstration is illustrated in Fig. 3a. In this configuration, the input RF signal is encoded onto the optical tone using a phase modulator. In the absence of the LO, the phase modulation alone does not drive an acoustic field in the PPER device, since a stimulated Brillouin process is driven by optical intensity and requires modulation of the optical power to drive acoustic fields^27^. However, the introduction of the optical LO produces intensity modulation through the beat note between the LO and the phase-modulation sideband (see inset (I) in Fig. 3a). By tuning the LO frequency, different spectral components of the phase modulation sideband participate in the signal transduction and get encoded onto the phase of the light in the ‘receive’ waveguide. The output light from the PPER device is demodulated and combined with the wideband signal from the input and the LO (see inset (II) in Fig. 3a) before detection, resulting in an RF signal with a notch response at the filter output.

We note that the detected RF signal at the filter output is determined by the same beat note that is driving the acoustic field, which is set by the LO. Hence, the output RF signal will be shifted in frequency compared to the input RF signal, such that the filtered frequency will always be at the Brillouin frequency (Ω_0_)^29^. Alternatively, this can be understood as shifting the input signal with respect to a notch filter, which remains centered around the Brillouin frequency. In our demonstration, the optical LO is synthesized from the same laser source, using an intensity modulator driven at frequency Ω_LO_ and a commercial optical bandpass filter, such that the LO is at optical frequency *ω*_LO_ = *ω*_1_ + Ω_LO_.

First, we demonstrate the shifting of the notch filter over a large spectral range. Figure 3b shows aggregated data from multiple measurements, in which the notch frequency is shifted in 2 GHz increments while maintaining its narrowband lineshape. Each trace was acquired by setting the LO frequency (Ω_LO_), which determines the notch frequency, and sweeping the input RF signal (Ω_RF_) over a 1 GHz range. The measurements show a flat RF response over the sweeping range and ~40 dB of signal suppression at the notch frequency, and an identical filter lineshape.

In all of the measurements, the output RF signal was measured around the Brillouin frequency (Ω_0_ = ∣Ω_RF_ − Ω_LO_∣), such that demodulation and detection are identical to the static filter case. For example, the notch response around 8.2 GHz (green trace in Fig. 3b) was obtained by setting the LO frequency at Ω_LO_/(2*π*) = 4 GHz, sweeping the input RF signal in the range Ω_RF_/(2*π*) = 7.7–8.7 GHz, and measuring the output RF signal in the range 3.7–4.7 GHz (centered around the Brillouin frequency Ω_0_/(2*π*) = 4.32 GHz). Hence, the tuning scheme demonstrated here performs down-conversion of the signal within the filtering process, which can be a useful feature in microwave-photonic applications that require frequency conversion^31–33^. Alternative schemes can be implemented to enable frequency-neutral filtering (see Supplement, Section 7 for an example).

Next, we proceed to show how the narrow bandwidth of the filter enables selective suppression of RF tones with ~MHz frequency resolution. We synthesize an input signal consisting of multiple RF tones separated in frequency by 5 MHz, seen in Fig. 3c. While keeping the input RF signal unchanged, we tune the LO such that the notch frequency is shifted, and selectively suppress specific frequency components without affecting nearby signals. Together, these demonstrations show wideband tunability while maintaining high resolution, a result of the combination of the photonic platform and the unique properties of acoustic waves.

### Microwave-photonic link performance

The viability of such new notch filtering schemes as the basis for impactful new technologies largely hinges on our ability to preserve signal fidelity within a microwave-photonic link. For this reason, we next investigate a full microwave-photonic link implementing a PPER-based notch filter, studying its noise and nonlinear properties, including link gain, noise figure, and dynamic range. We characterize the RF link using the experimental scheme shown in Fig. 1b, with an additional optical amplifier (EDFA) at the output of the PPER device to offset fiber-chip coupling losses. The RF link measurements were performed with an optical power of *P*^(B)^ = 79 mW on the detector, and we estimate the optical power in the ‘emit’ waveguide of the PPER device at *P*^(E)^ = 50 mW. The device used for the measurement was a phononic-crystal PPER (see Fig. 2d) with an active length of *L* = 17 mm. In our measurements, the dominant noise source was amplified spontaneous emission from the optical amplifiers, with a measured noise floor of *N* = −125.1 dBm/Hz.

**Fig. 4:**
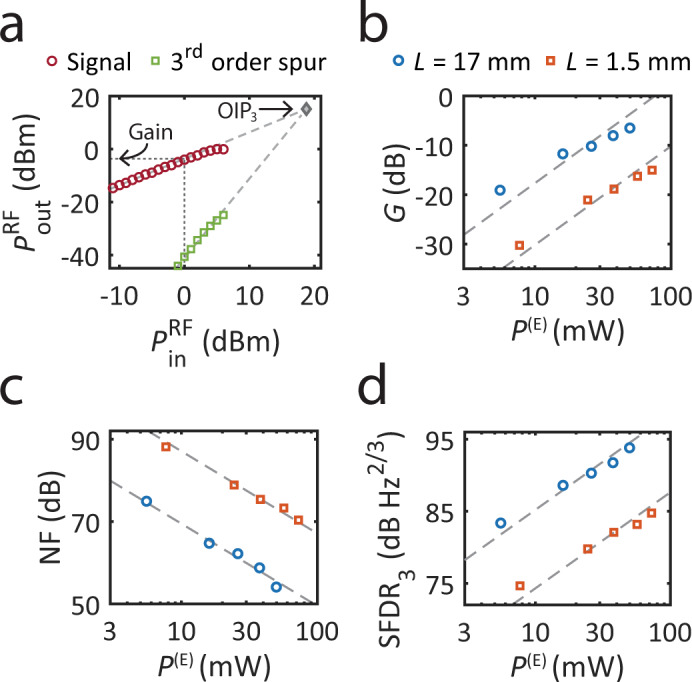
Microwave-photonic link performance. **a** Microwave-photonic link measurements; RF output power as a function of RF input power, showing the signal the third-order spur. Extrapolated linear trends and the third-order intercept point are shown for reference. **b** RF link gain as a function of optical power in the ‘emit’ waveguide. The measurements were performed using two different PPER devices, with active lengths of *L* = 17 mm (blue) and *L* = 1.5 mm (red). **c** Corresponding noise figure and **d** third-order spur-free dynamic range. The intermodulation SFDR_3_ is 3.2 dB Hz^2/3^ lower than the results shown here, which were measured using the third harmonic.

Next, we measure the linear and nonlinear properties of the microwave-photonic link implementing a narrowband notch filter. When operating a notch filter, signals of interest are in the filter pass-band; hence, we characterize the link properties outside the notch bandwidth. First, an input RF tone at frequency Ω_in_ = Ω_0_ + *δ*Ω (where *δ*Ω = 30 MHz and Ω_0_ is the notch frequency) is injected into the link input, and the RF power at the link output is measured at the same RF frequency. The output RF power shows a linear response with input RF power, as seen in Fig. 4a. The RF link gain was measured to be *G* = −3.6 dB, such that the microwave-photonic link has a noise figure of NF = 52.5 dB (where the input noise is assumed to be *N*_in_ = *k*_B_*T* = −174 dBm/Hz).

At high input RF powers, the microwave-photonic link exhibits a nonlinear response, such that the output power deviates from the small-signal linear scaling with input RF power. First, we measure the compression of the output power, yielding a linear dynamic range of CDR_1dB_ = 125.6 dB Hz. To characterize the spur-free dynamic range of the microwave-photonic link, we measure the propagation of a third harmonic distortion through the link. More specifically, the input RF signal frequency was set to Ω_in_ = (Ω_0_ + *δ*Ω)/3, and the RF power at frequency Ω_out_ = 3Ω_in_ was measured at the link output. As can be seen in Fig. 4a, the output power follows a cubic trend, as expected from a third harmonic measurement. Extrapolating the linear and nonlinear measurements, we calculate a spur-free dynamic range of SFDR_3_ = 93.6 dB Hz^2/3^. The corresponding inter-modulation SFDR_3_, such as would be obtained from a two-tone measurement, is 3.2 dB Hz^2/3^ lower^34^. A summary of the measured RF link parameters is presented in Table 1.

**Table 1:** Microwave-photonic link parameters, corresponding to the data presented in Fig. 4a.

Measured parameter	Description
*G* = −3.6 dB	RF link gain
*N* = −125.1 dBm/Hz	Noise floor
NF = 52.5 dB	Noise figure
OIP_3_ = 15.1 dBm	Output intercept point
SFDR_3_ = 93.6 dB Hz^2/3^	Spur-free dynamic range
${P}_{{{{{{{\rm{in}}}}}}}}^{1{{{{{{\rm{dB}}}}}}}}=4.1$ dBm	1 dB compression point
CDR_1dB_ = 125.6 dB Hz	Linear dynamic range

The noise figure is given by NF = *N* − *G* − *N*_in_ (with *N*_in_ = *k*_B_*T*); spur-free dynamic range is given by SFDR_3_ = (2/3)(OIP_3_ − *N*); linear dynamic range is given by ${{{{{{{\rm{CDR}}}}}}}}_{1{{{{{{\rm{dB}}}}}}}}={P}_{{{{{{{\rm{in}}}}}}}}^{1{{{{{{\rm{dB}}}}}}}}+G-N$. Intermodulation OIP_3_ and SFDR_3_ values are 4.8 dBm and 3.2 dB Hz^2/3^ lower than presented here, respectively.

Looking beyond this first proof-of-principle demonstration, it is instructive to investigate how the device parameters impact various aspects of link performance to guide future designs and realize improved performance. The acousto-optic interaction strength in the PPER device is determined by a product of the optical power in the ‘emit’ waveguide (*P*^(E)^), the active-region length (*L*), and the Brillouin gain (*G*_B_)^26,35^. This figure of merit plays a key role in the performance of microwave-photonic links using a PPER device, such as the notch filters we are studying here.

To demonstrate the potential of the presented filtering scheme, we show how the link parameters scale with optical power and device length. First, we repeat the RF link measurements using different optical powers in the ‘emit’ waveguide of the PPER device. By tuning the optical power injected into the ‘emit’ waveguide of the device, we can see the enhancement of the link gain, which scales as $G\propto {({G}_{{{{{{{\rm{B}}}}}}}}{P}^{{{{{{{\rm{(E)}}}}}}}}L)}^{2}$, the reduction of the noise figure, which scales as ${{{{{{\rm{NF}}}}}}}\propto {({G}_{{{{{{{\rm{B}}}}}}}}{P}^{{{{{{{\rm{(E)}}}}}}}}L)}^{-2}$ and the improvement in dynamic range, which scales as $\,{{{{{{\rm{SFDR}}}}}}}\propto {({G}_{{{{{{{\rm{B}}}}}}}}{P}^{{{{{{{\rm{(E)}}}}}}}}L)}^{4/3}$, with higher optical power, as seen in Fig. 4b–d. Next, we illustrate the scaling of the RF link parameters with device length, by repeating the measurements using a shorter PPER device with an active-region length of *L* = 1.5 mm. As can be seen from Fig. 4b–d, the shorter device yields an RF link with lower gain, higher noise figure, and a smaller dynamic range. This degradation in performance is expected since the Brillouin interaction strength (*G*_B_*P*^(E)^*L*) is ~10 times weaker for the shorter device (see Supplement, Sections 2 and 5).

## DISCUSSION

In this work, we have demonstrated a tunable microwave-photonic notch filter that utilizes Brillouin-based acoustic signal transduction to realize high-resolution microwave-photonic notch filters, which would otherwise be very difficult to create using all-optical methods. The photonic-phononic emit-receive (PPER) device geometry used here leverages standard SOI process, and waveguide geometries that are common to a variety of silicon-photonics foundries. Moreover, this filtering scheme eliminates the need for exotic nonlinear materials, circulators, isolators, advanced modulation schemes, and pump-rejection filters—which previous Brillouin-based microwave-photonic filters have required—lowering the barrier towards full integration on-chip and enabling the realization of more sophisticated microwave-photonic signal processing capabilities.

**Fig. 5:**
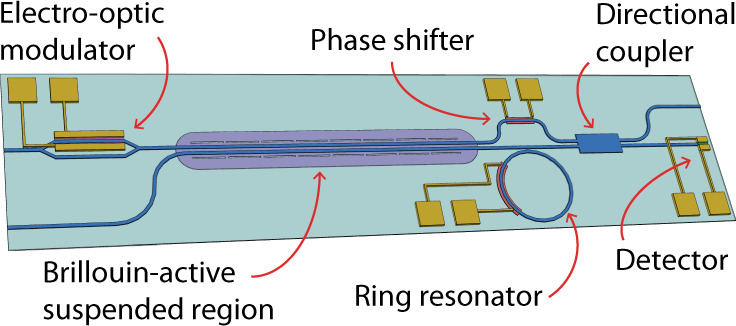
Fully integrated microwave-photonic notch filter. Schematic illustration of an integrated PPER-based notch filter, including an electro-optic modulator, a Brillouin-active suspended region (PPER device), and a detector. Other integrated components can include ring resonators, phase shifters, and directional couplers. In this example, the ‘emit' waveguide (upper waveguide in the Brillouin-active region) serves as the upper arm of the interferometer in Fig. 1 (see Supplement, Section 2).

Since our Brillouin-based device is fabricated in silicon, using foundry-compatible waveguide geometries, this device strategy permits us to leverage well-established manufacturing techniques within the rapidly growing field of integrated photonics^36^. By integrating modulators^37,38^, phase shifters^39^, optical attenuators^40^, filters^41^, and detectors^42–44^, the PPER-based filter could be fully implemented on-chip. As an illustration, Fig. 5 shows one implementation of an integrated notch filter that could readily be fabricated in a variety of silicon photonics facilities^45,46^, for example, by using a local backside release^47^. Furthermore, since the rejection (57 dB) of the demonstrated filter was limited by the stability of discrete fiber-based components used for this laboratory proof-of-principle, integrating the filter on-chip would likely yield higher stop-band rejection.

Building on the measured scaling of the link performance (link gain, noise figure, and spur-free dynamic range), and with further RF link analysis, we see that this microwave-photonic link can be drastically improved with better integration and the use of a state-of-the-art modulator. In our proof-of-principle demonstration, optical amplifiers (EDFAs)—used to offset fiber-chip coupling losses—account for the primary source of excess noise that degraded the link performance. However, a fully integrated PPER-based filter operating under the same conditions would not require such amplification, yielding an improvement of the noise figure by ~30 dB and an improvement of the dynamic range of the RF link by ~20 dB (see Supplement, Section 5). Furthermore, the use of a modulator with a low half-wave voltage^48,49^ will further improve the noise figure and dynamic range. For example, using state-of-the-art modulators and increasing the device *G*_B_*P*^(E)^*L* product would permit noise figures below 10 dB and a dynamic range larger than 110 dB Hz^2/3^ (see Supplement, Section 5 for further details). It is important to note that noise from the Brillouin process does not impact the link performance. Namely, thermal-mechanical noise—a major challenge in optomechanics-based bandpass filtering schemes—is centered at the rejection band of the filter. Thus, the Brillouin process only contributes noise at the notch frequency (i.e., at the Brillouin frequency^26,29^), and does not affect the filter pass-band.

In practical RF systems, there may be multiple RF interferers, requiring more complex notch filtering schemes to enable low-noise operation. Such complex microwave-photonic systems could be implemented using fully integrated Brillouin-based devices—leveraging the ability to combine a large number of devices in different configurations within a small footprint—with scalability that would be very difficult to achieve using discrete components^50,51^. For example, multiple filters could be cascaded in series, with minimal signal degradation thanks to the low-loss waveguides (~0.2 dB/cm) used in the devices^11,26,28^. Combining the scalability of integrated photonics with the multi-port PPER-based filter enables the design of versatile signal-processing schemes in a variety of configurations (see Supplement, Sections 6–8).

In summary, we have presented a microwave-photonic notch filter—based on a multi-port Brillouin-active PPER device—that produces high signal rejection (~60 dB) within a narrow spectral band (~MHz). We have demonstrated that this microwave-photonic system enables tunability of the notch frequency over a wide spectral band. These multi-port Brillouin-active devices were fabricated using standard silicon-photonics fabrication processes and waveguide dimensions, which bodes well for large-scale integration^47^. Through earlier studies, such suspended Brillouin-active waveguide devices have been shown to yield repeatable and robust performance over long periods of time^29^. As photonic technologies progress towards the integration of multiple components on-chip, microwave-photonic circuits of the type demonstrated here could be a step towards fully integrated, CMOS-compatible microwave-photonic signal-processing systems.

## Methods

### Device fabrication

The PPER device using slots for acoustic confinement was fabricated at the MESA facilities of Sandia National Laboratories. The fabrication steps utilize standard silicon-photonics photolithography processing methods, followed by a hydrofluoric acid etch to remove the oxide under-cladding. The devices utilizing phononic crystals used in this work were fabricated using standard electron-beam lithography. For details about the fabrication steps, see the Supplementary Information of ref. ^29^.

### Experiment

Both experiments used lasers operating around 1550 nm. All devices use integrated grating couplers to couple light on and off the chip.

## Supplementary information

Supplementary Information

## Data Availability

Data supporting the findings of this study are available upon request.
