# Supplementary material for: Narrowband microwave-photonic notch filters using Brillouin-based signal transduction in silicon
Source: Nat Commun. 2022 Apr 11;13:1947. doi: 10.1038/s41467-022-29590-0 (PMC9001665; doi:10.1038/s41467-022-29590-0)
Supplement: Supplementary file 1 — Supplementary Information [file 41467_2022_29590_MOESM1_ESM.pdf]

Supplementary Information: Narrowband microwave-photonic notch filters using Brillouin-based signal transduction in silicon

Shai Gertler,[1,*] Nils T. Otterstrom,[1,2] Michael Gehl,[2] Andrew L. Starbuck,[2] Christina M. Dallo,[2] Andrew T. Pomerene,[2] Douglas C. Trotter,[2] Anthony L. Lentine,[2] and Peter T. Rakich[1,†]

[1] *Department of Applied Physics, Yale University, New Haven, CT 06520, USA*

[2] *Photonic and Phononic Microsystems, Sandia National Laboratories, Albuquerque, New Mexico 87185, USA*

Contents

I. Survey of integrated microwave-photonic notch filters 2

II. PPER-based notch filtering 2
Using the 'emit' waveguide output for filtering 5

III. Second-order filters 6

IV. Devices used in the experiments 8

V. RF link properties 9

VI. Frequency response manipulation 11
Notch-frequency selection 11
Inverting the frequency response 13

VII. Alternative notch-filtering implementations 14
Frequency-neutral tunable filter 14
Using two photo-detectors 15
Signal interference in the optical domain 17

VIII. Filter arrays 20

References 22

* shai.gertler@yale.edu
† peter.rakich@yale.edu

I. SURVEY OF INTEGRATED MICROWAVE-PHOTONIC NOTCH FILTERS

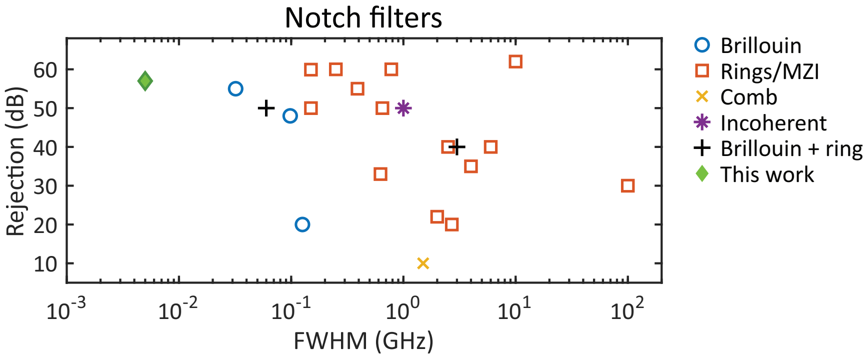

FIG. S1: Summary of recently demonstrated integrated microwave-photonic notch filters, showing filter rejection and spectral resolution (FWHM: full-width at half-maximum). Surveyed work includes Brillouin-based filtering [1–3], filters using ring resonators and interferometers [4–18], comb-generation and spectral shaping [19], incoherent multi-tap filtering [20], and Brillouin scattering combined with resonators [21, 22].

II. PPER-BASED NOTCH FILTERING

We start by describing how the interferometric scheme utilizing a PPER device, illustrated in Fig. S2, yields a microwave-photonic notch-filtering operation. First, the input RF signal is encoded on an optical carrier with frequency ω_1 using an intensity modulator, yielding a field amplitude given by [23]

$$E^{\mathrm{IM}}(t) = \sqrt{\frac{\widetilde{P}^{(\mathrm{A})}}{2}} e^{-i\omega_1 t}\left(e^{i\theta} + \sum_n J_n\left(\frac{\pi V_{\mathrm{in}}}{V_\pi}\right) e^{-in\Omega t}\right). \tag{1}$$

Here, $\widetilde{P}^{(\mathrm{A})}$ denotes the optical power, the input voltage oscillates at frequency Ω with an amplitude V_{in}, and the half-wave voltage of the modulator is denoted V_π. The angle θ is determined by the biasing point of the modulator, and for the rest of our analysis, we will assume that it is biased at quadrature (i.e., $\theta = \pi/2$). The field is split using a directional coupler, and one branch is directed into the 'emit' waveguide of a PPER device, while the other bypasses the device completely, and is directed to a photodetector. A separate laser source with frequency ω_2 is used for the 'receive' path of the PPER, and at the output of the PPER device this results in a phase-modulated signal [24]

$$E^{\mathrm{PPER}}(t) = \sqrt{\widetilde{P}^{(\mathrm{B})}}\; e^{-i\omega_2 t}\; e^{i\beta_{\mathrm{in}}\cos\left(\Omega t - \phi\right)}. \tag{2}$$

Here, $\widetilde{P}_B$ is the 'receive' optical power, and the modulation index is given by

$$\beta_{\mathrm{in}} = G_{\mathrm{B}} P^{(\mathrm{E})} L J_1\left(\frac{\pi V_{\mathrm{in}}}{V_\pi}\right) \frac{\Gamma}{2} \left|\chi(\Omega)\right|, \tag{3}$$

where G_{B} is the Brillouin gain, $P^{(\mathrm{E})}$ is the power in the 'emit' waveguide, L is the length of the active region of the device, and $J_n(\cdot)$ is an nth order Bessel function. Above, the phonon lifetime is given by Γ, $\chi(\Omega)$ is the acoustic frequency response, and we have denoted the phase $\phi(\Omega) = \arg(\chi(\Omega))$. Using the Jacobi-Anger expansion, Eq. (2) can be expressed as

$$E^{\mathrm{PPER}}(t) = \sqrt{\widetilde{P}^{(\mathrm{B})}}\; e^{-i\omega_2 t} \sum_n i^{-n} J_{-n}\left(\beta_{\mathrm{in}}\right) e^{-in(\Omega t - \phi)}. \tag{4}$$

The phase of the optical field from Eq. (4) is demodulated using an optical filter, such that we are left with an optical carrier and a single sideband

$$\bar{E}^{\mathrm{PPER}}(t) = \sqrt{\widetilde{P}^{(\mathrm{B})}} e^{-i\omega_2 t}\left(J_0\left(\beta_{\mathrm{in}}\right) + iJ_1\left(\beta_{\mathrm{in}}\right) e^{-i(\Omega t - \phi)}\right). \tag{5}$$

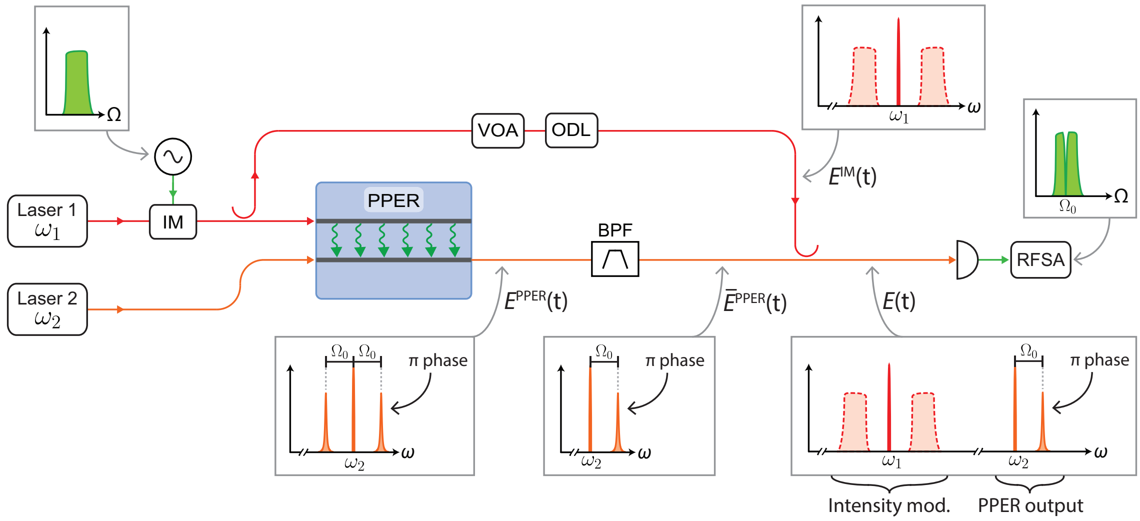

FIG. S2: The PPER-based notch filter scheme; the interference between the signal from the intensity modulator and the demodulated PPER output results in cancellation of the RF signal at the Brillouin frequency (Ω_0). IM: intensity modulator, VOA: variable optical attenuator, ODL: optical, delay line, BPF: optical bandpass filter, RFSA: RF spectrum analyzer.

In our analysis, we have assumed that the demodulation is performed using an ideal filter, such that there is no attenuation for the transmitted carrier and sideband, while all other optical sidebands are completely suppressed.

The demodulated PPER output field is directed to the photodetector, such that the total detected field is given by

$$\begin{aligned} E(t) = \sqrt{\frac{P^{(\mathrm{A})}}{2}} e^{-i\omega_1\left(t-\tau^{(\mathrm{A})}\right)} \left(i + \sum_n J_n \left(\frac{\pi V_{\mathrm{in}}}{V_\pi} \right) e^{-in\Omega\left(t-\tau^{(\mathrm{A})}\right)} \right) \\ + i\sqrt{P^{(\mathrm{B})}} e^{-i\omega_2\left(t-\tau^{(\mathrm{B})}\right)} \left(J_0\left(\beta_{\mathrm{in}}\right) + iJ_1\left(\beta_{\mathrm{in}}\right) e^{-i\left(\Omega\left(t-\tau^{(\mathrm{B})}\right)-\phi\right)} \right), \end{aligned} \tag{6}$$

where the first line represents the optical field amplitude from the intensity modulator (bypassing the PPER device) and the second line is the demodulated PPER output. Here, $\tau^{(\mathrm{A})}$ and $\tau^{(\mathrm{B})}$ are the time delays experienced by the fields in each of the optical paths. The relation between $\{\widetilde{P}^{(\mathrm{A})}, \widetilde{P}^{(\mathrm{B})}\}$ and $\{P^{(\mathrm{A})}, P^{(\mathrm{B})}\}$ is determined by the splitting ratio of the directional coupler used to combine the signals before detection (for example, a 50:50 coupler would yield $P^{(\mathrm{A})} = \widetilde{P}^{(\mathrm{A})}/2$ and $P^{(\mathrm{B})} = \widetilde{P}^{(\mathrm{B})}/2$), and the factor i is a result of the phase imparted in the coupling. The photocurrent at the detector is given by $I = \eta P$, where η is the detector responsivity, and the fields are normalized such that $P = |E|^2$. Isolating the current oscillating at frequency Ω, we are left with

$$I^{(\Omega)}(t) = 2\eta \left[P^{(\mathrm{B})} J_0\left(\beta_{\mathrm{in}}\right) J_1\left(\beta_{\mathrm{in}}\right) \sin\left(\Omega\left(t-\tau^{(\mathrm{B})}\right) - \phi \right) - P^{(\mathrm{A})} J_1\left(\frac{\pi V_{\mathrm{in}}}{V_\pi} \right) \sin\left(\Omega\left(t-\tau^{(\mathrm{A})}\right) \right) \right]. \tag{7}$$

We note that the two optical wavelengths from the two lasers are chosen such that the beat note between the carriers is well beyond the detector bandwidth, and does not contribute to the RF signal. This is easily implemented, as a difference as small as $\Delta\lambda = 1$ nm (where $\Delta\lambda = |2\pi c/\omega_1 - 2\pi c/\omega_2|$) corresponds to $\Delta\omega/(2\pi) \approx 125$ GHz (where $\Delta\omega = |\omega_1 - \omega_2|$), well beyond the bandwidth of typical detectors.

In the small-signal limit (when $V_{\mathrm{in}} \ll V_\pi$), we can expand the Bessel functions to first order ($J_0(x) \approx 1, J_1(x) \approx x/2$), and ignoring an overall phase, this leaves us with the RF signal

$$I^{(\Omega)}(t) = \eta \left(\frac{\pi V_{\mathrm{in}}}{V_\pi} \right) \left[P^{(\mathrm{A})} \sin\left(\Omega t\right) - \left(\frac{1}{2} P^{(\mathrm{B})} G_{\mathrm{B}} P^{(\mathrm{E})} L \frac{\Gamma}{2} \left|\chi(\Omega)\right| \right) \sin\left(\Omega t + \Omega\Delta\tau - \phi \right) \right], \tag{8}$$

where we have substituted β_{in} from Eq. 3, and denoted the time difference betwen the two optical paths as $\Delta\tau = \tau^{(\mathrm{A})} - \tau^{(\mathrm{B})}$. Rearranging this expression, we arrive at

$$I^{(\Omega)}(t) = \eta \left(\frac{\pi V_{\mathrm{in}}}{V_\pi} \right) \mathrm{Im}\left[e^{-i\Omega t} \left(P^{(\mathrm{A})} - \frac{1}{2} P^{(\mathrm{B})} G_{\mathrm{B}} P^{(\mathrm{E})} L \frac{\Gamma}{2} \left|\chi(\Omega)\right| e^{-i(\Omega\Delta\tau - \phi)} \right) \right]. \tag{9}$$

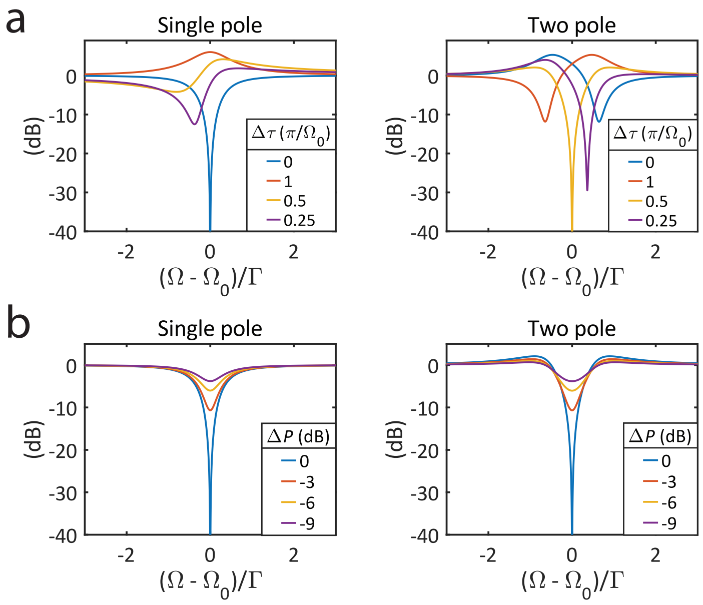

FIG. S3: **(a)** Left: Calculated frequency response of the notch filter scheme for the case of a single-pole filter, showing different values of time-delay between the two interferometer paths (see Eq. (9)). Right: Equivalent calculation for the case of a two-pole filter. **(b)** Left: Calculated frequency response for different cases of power difference between the two signals. To achieve perfect signal cancellation, the two signals need to be power balanced, i.e., $\Delta P = 0$. Right: Equivalent calculation for the case of a two-pole filter.

We note that $P^{(\mathrm{A})}$ and $P^{(\mathrm{B})}$ are the optical power on the detector from the unfiltered interferometer path and the PPER path, respectively, while $P^{(\mathrm{E})}$ represents the optical power in the 'emit' waveguide of the PPER device. From Eq. (9) we can see how complete cancellation of the RF signal can be achieved at the Brillouin frequency (Ω_0) by setting the power ($P^{(\mathrm{A})}$) and time delay ($\Delta\tau$) such that $P^{(\mathrm{A})} = P^{(\mathrm{B})} G_\mathrm{B} P^{(\mathrm{E})} L \Gamma |\chi(\Omega_0)|/4$ and $\Omega_0 \Delta\tau = \phi(\Omega_0) + 2\pi m$ (where m is an integer), as seen in Fig. S3.

The conditions for signal cancelation at the notch frequency are dependent on the frequency response of the PPER device $\chi(\Omega)$. We begin by examining the case of a single-pole PPER response, given by [25]

$$\chi(\Omega) = \frac{1}{i(\Omega_0 - \Omega) + \Gamma/2}, \tag{10}$$

such that at the Brillouin frequency (Ω_0) we have

$$|\chi(\Omega_0)| = \frac{2}{\Gamma}, \qquad \phi(\Omega_0) = 0. \tag{11}$$

From Eq. (9) we see that in order of achieve signal cancellation at Ω_0 we need to set $P^{(\mathrm{A})} = P^{(\mathrm{B})} G_\mathrm{B} P^{(\mathrm{E})} L/2$ and $\Delta\tau = 2\pi m/\Omega_0$, yielding

$$I^{(\Omega)}(t) = \eta \left(\frac{\pi V_\mathrm{in}}{V_\pi}\right) \left(\frac{1}{2} P^{(\mathrm{B})} G_\mathrm{B} P^{(\mathrm{E})} L\right) \mathrm{Im}\left[e^{-i\Omega t} \underbrace{\left(1 - \frac{\Gamma}{2}\chi(\Omega) e^{-i(2\pi m \Omega/\Omega_0)}\right)}_{\xi(\Omega)}\right]. \tag{12}$$

Here, we have defined the frequency response of the notch filter as $\xi(\Omega)$. The calculated filter lineshape is consistent with measurements, as seen in Fig. S4.

Finally, we analyze the DC photocurrent at the detector, which is important for designing practical systems that have power-handling limitations, and for estimating the noise floor of the microwave-photonic link. Calculating the DC terms of the photocurrent, we have

$$I^{(\mathrm{DC})} = \eta\Big(P^{(\mathrm{A})} + P^{(\mathrm{B})}\left[J_0^2(\beta_\mathrm{in}) + J_1^2(\beta_\mathrm{in})\right]\Big) \approx \eta\Big(P^{(\mathrm{A})} + P^{(\mathrm{B})}\Big), \tag{13}$$

where in the last step we used a small-signal approximation, consistent with our earlier analysis.

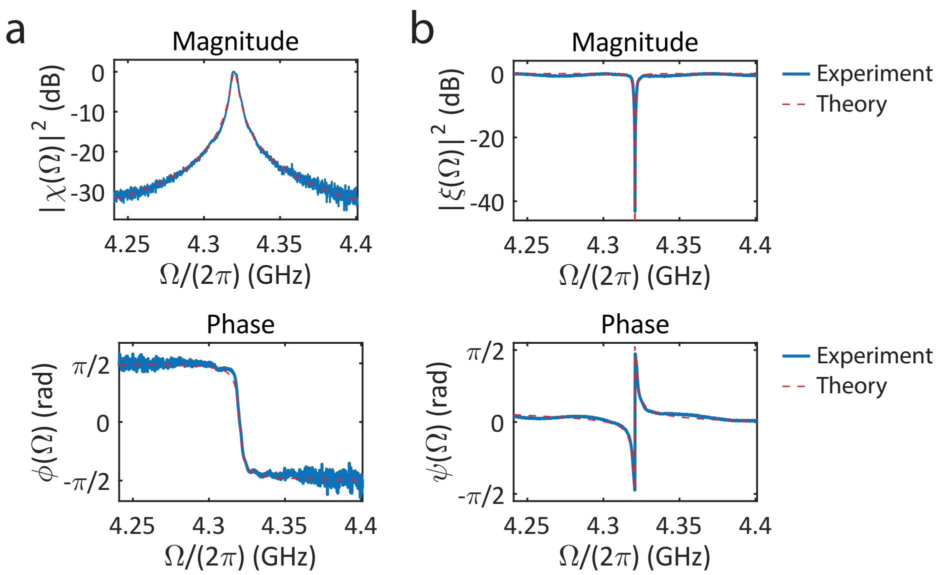

FIG. S4: **(a)** Measured frequency response of a single-pole PPER device. Top: magnitude; Bottom: phase. **(b)** The corresponding notch filter response. The theoretical trends follow Eqs. (10) and (12).

Using the 'emit' waveguide output for filtering

In our demonstrations, the upper arm of the interferometer from Fig. S2 (going through the VOA and ODL) bypassed the PPER altogether. Alternatively, it is possible to use the 'emit' waveguide as the upper interferometer arm, as illustrated in Fig. S5. Within the PPER, the intensity-modulated light in the 'emit' waveguide drives the phonon field, resulting in the phase modulation of light propagating in the 'receive' waveguide. The forward Brillouin process in the device also results in the phase modulation of the light in the emit waveguide, however, it does not affect the intensity modulation of the light [25].

At the output of the 'emit' waveguide, the optical field amplitude is given by [24]

$$E^{(\mathrm{E})}(t) = \sqrt{\frac{P^{(\mathrm{E})}}{2}} e^{-i\omega_1 t} \left(i + \sum_n J_n \left(\frac{\pi V_{\mathrm{in}}}{V_\pi} \right) e^{-in\Omega t} \right) e^{i\beta_{\mathrm{in}} \cos\left(\Omega t - \phi\right)}, \tag{14}$$

where we have assumed that the intensity modulator at the RF link input is biased at quadrature. When combining with the light exiting the 'receive' waveguide (after phase demodulation), we have

$$\begin{aligned} E^{(\mathrm{E})}(t) = \sqrt{\frac{P^{(\mathrm{A})}}{2}} e^{-i\omega_1 \left(t - \tau^{(\mathrm{A})}\right)} \left(i + \sum_n J_n \left(\frac{\pi V_{\mathrm{in}}}{V_\pi} \right) e^{-in\Omega\left(t-\tau^{(\mathrm{A})}\right)} \right) e^{i\beta_{\mathrm{in}} \cos\left(\Omega\left(t-\tau^{(\mathrm{A})}\right) - \phi\right)} \\ + i\sqrt{P^{(\mathrm{B})}} e^{-i\omega_2\left(t-\tau^{(\mathrm{B})}\right)} \left(J_0\left(\beta_{\mathrm{in}}\right) + iJ_1\left(\beta_{\mathrm{in}}\right) e^{-i\left(\Omega\left(t-\tau^{(\mathrm{B})}\right)-\phi\right)} \right). \end{aligned} \tag{15}$$

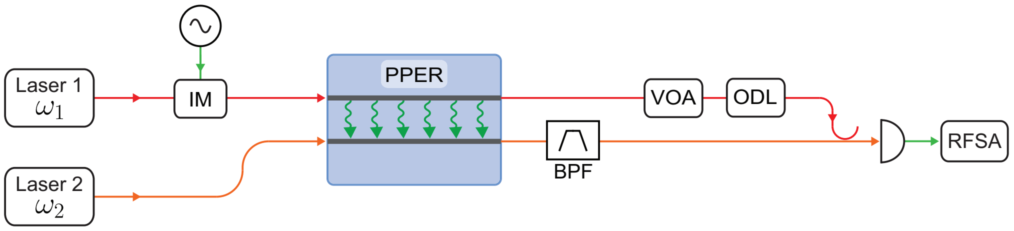

FIG. S5: PPER-based notch filtering scheme, using the 'emit' waveguide as one of the interferometer paths. IM: intensity modulator, VOA: variable optical attenuator, ODL: optical, delay line, BPF: optical bandpass filter, RFSA: RF spectrum analyzer.

Here, the optical power $P^{(A)}$ denotes the power from the emit waveguide at the detector, taking into account the directional coupler that combines the two interferometer paths, as well as additional attenuation set by the variable optical attenuator (VOA). The time delays $\tau^{(A)}$ and $\tau^{(B)}$ are the delays experienced by each of the optical waves.

We can see that Eq. (15) is similar in form to Eq. (6), except for the phase modulation term multiplying the field from the upper interferometer path. Since we are assuming that the two optical frequencies are well separated (i.e., $|\omega_1 - \omega_2| \gg \Omega$), when we calculate the photocurrent ($I \propto |E|^2$), we can neglect the cross-term between the two fields from Eq. (15), and the phase modulation term will vanish when squaring the field. The resulting photocurrent will be identical to that from Eq. (7), yielding the notch filter response when balancing the interferometer (i.e., setting the required power and time delay, as discussed earlier).

III. SECOND-ORDER FILTERS

The multi-port PPER scheme can enable the design of devices with a multi-pole frequency response, by utilizing multiple coupled acoustic modes in the transduction process [25]. Using such multi-pole devices within the notch-filtering scheme produces microwave-photonic filters with a multi-pole frequency response.

Next, we proceed to demonstrate the use of a two-pole PPER device, such as was described in Ref. [26], within the notch- filtering scheme. The device used in the measurements is shown in Figs. S6(a), where a phononic-crystal region between the two waveguides enables controlled coupling of two Brillouin-active acoustic modes. The frequency response obtained by such a device exhibits a two-pole lineshape, given by [25]

$$\chi^{(\text{2-pole})}(\Omega) = \frac{1/2}{i(\Omega_0 - \mu - \Omega) + \Gamma/2} - \frac{1/2}{i(\Omega_0 + \mu - \Omega) + \Gamma/2}, \tag{16}$$

where μ is the coupling rate between the two acoustic modes taking part in the filtering process. The resulting lineshape has a sharp frequency roll-off compared to the typical Lorentzian response obtained from a single acoustic resonance. At the Brillouin frequency we have

$$\left|\chi^{(\text{2-pole})}(\Omega_0)\right| = \frac{\mu}{\mu^2 + (\Gamma/2)^2}, \qquad \phi^{(\text{2-pole})}(\Omega_0) = \frac{\pi}{2} + \arg(\mu) - \arg\left(\mu^2 + (\Gamma/2)^2\right). \tag{17}$$

For this analysis we will assume $\mu = \Gamma/2$, where signal cancellation at the notch frequency requires setting the optical power ($P^{(A)}$) and the time delay ($\Delta\tau$) such that $P_A = P_B G_\text{B} P_E L/4$ and $\Delta\tau = (\pi/2 + 2\pi m)/\Omega_0$. Substituting these parameters into Eq. (9) yields

$$I^{(\Omega)}(t) = \eta\left(\frac{\pi V_\text{in}}{V_\pi}\right)\left(\frac{1}{4}P_B G_\text{B} P_E L\right)\text{Im}\left[e^{-i\Omega t}\underbrace{\left(1 - \frac{\Gamma}{2}\chi^{(\text{2-pole})}(\Omega)e^{-i\left[(\pi/2+2\pi m)(\Omega/\Omega_0)\right]}\right)}_{\xi^{(\text{2-pole})}(\Omega)}\right], \tag{18}$$

where we have identified the frequency response of the two-pole notch filter $\xi^{(\text{2-pole})}(\Omega)$, showing signal suppression at $\Omega = \Omega_0$.

Experimentally, we use a two-pole PPER device with four lines of phononic-crystal holes (unit cells) between the two waveguides (see Fig. S6(a)), yielding the frequency response seen in Fig. S6(b). The device produces a two-pole response at frequency $\Omega_0/(2\pi) = 4.2$ GHz, with a FWHM of 11.2 MHz, and strong suppression of acoustic modes that are outside the phononic crystal stop-band. When using the two-pole device within the notch-filter scheme, as shown in the bottom panels of Figs. S6(b) and S6(c), we achieve a notch filter with FWHM of 7.3 MHz, signal suppression of 46 dB at the notch frequency (Ω_0), and less than 1 dB of ripple. The measured filter lineshapes are consistent with theory (Eqs. (16) and (18)), as seen in Fig. S7.

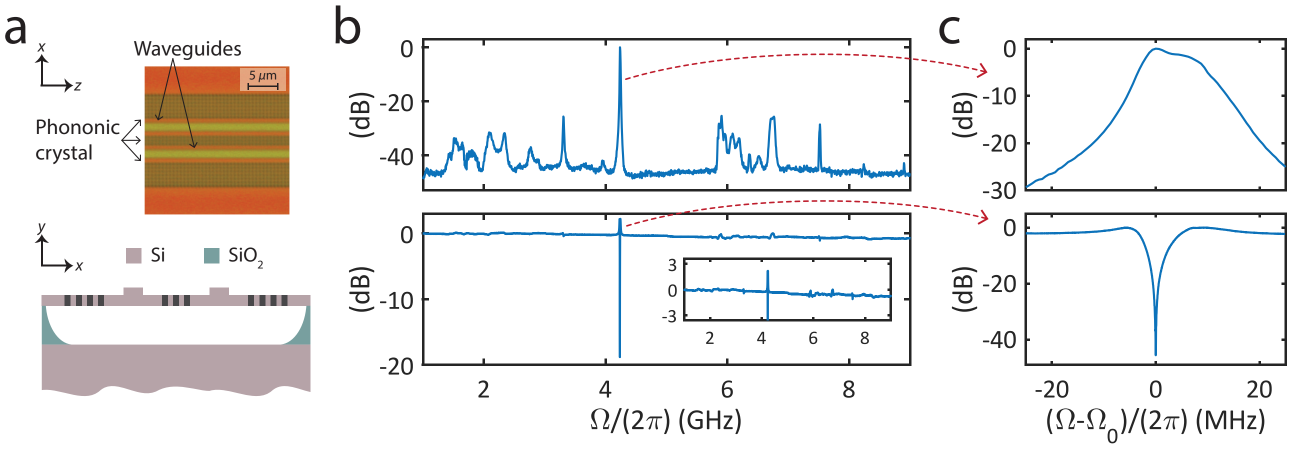

FIG. S6: **(a)** Top: Top-down micrograph of a two-pole PPER device, utilizing a phononic crystal structure. Bottom: Illustration of the device cross section. **(b)** Top: measured frequency response of the PPER device (top) and corresponding notch filter (bottom). Inset: magnified view of spurious peaks. **(c)** Magnified view of the frequency response around $\Omega_0 = 4.2$ GHz.

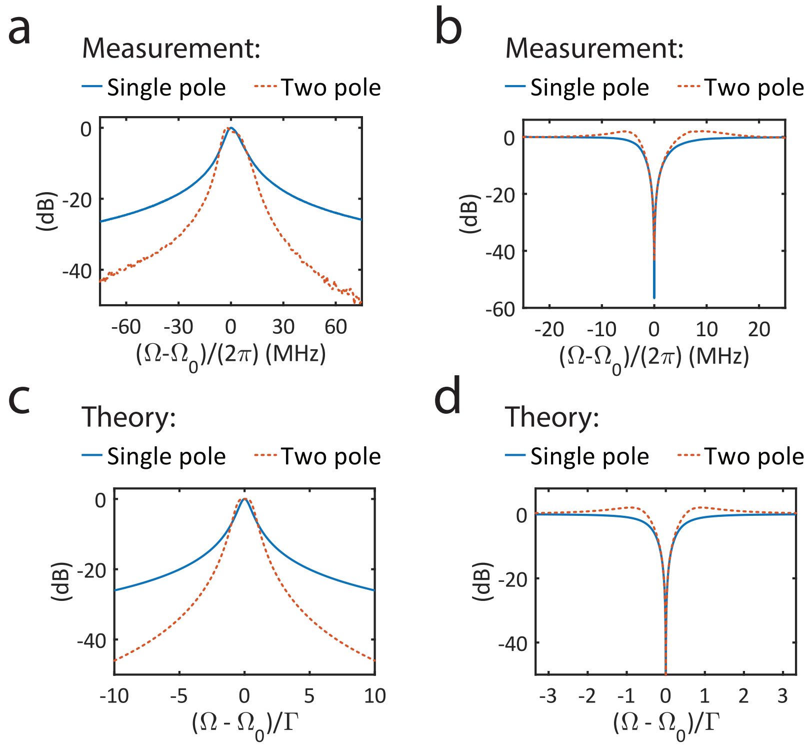

FIG. S7: **(a)** Measured frequency response of a single-pole PPER (blue) and a two-pole PPER (red, dashed) device. **(b)** Measurements of the corresponding notch filter responses. **(c)** Calculation of the expected frequency response, following Eqs. (10) and (16). **(d)** Calculation of the frequency response of the corresponding notch filters, using Eqs. (12) and (18). In the calculations, we have assumed an acoustic Q-factor of 1000, a time delay with $m = 0$, and for the two-pole filter case, we have used $\mu = \Gamma/2$.

IV. DEVICES USED IN THE EXPERIMENTS

The PPER device using slots for acoustic confinement, corresponding to the data shown in Figs. 2(b) and 2(c) of the main text, was fabricated at the Sandia Laboratory MESA facilities. The fabrication steps utilize standard silicon-photonics photolithography processing methods, followed by a hydrofluoric acid etch to remove the oxide under-cladding. The widths of the two waveguides are 1 μm and 0.85 μm, the distance between the slots is 5 μm, and the Brillouin-active length of the device is $L = 1.5$ mm. The device geometry is illustrated in Fig. S8(a).

The devices utilizing phononic crystals used in this work, seen in Figs. S8(b) and S8(c), were fabricated using standard electron-beam lithography. For details about the fabrication steps, see the Supplementary Information of Ref. [26].

The single-pole PPER device, corresponding to the data shown in Figs. 2(e) and 2(f) of the main text, is illustrated in Fig. S8(b). The widths of the two waveguides are 1 μm and 0.9 μm, the distance between the phononic crystal regions is 5 μm, and the Brillouin-active length of the device is $L = 1.7$ cm. Each phononic crystal region is a cubic lattice of air holes, with a pitch of 600 nm and a hole diameter of 500 nm, with seven rows of holes on either side of the waveguide.

The two-pole PPER device, corresponding to the data shown in Figs. S6(b) and S6(c) is illustrated in Fig. S8(c). The width of both waveguides is 1 μm, the distance between the phononic crystal regions is 3 μm, and the Brillouin-active length of the device is $L = 1.7$ cm. Each phononic crystal region is a cubic lattice of air holes, with a pitch of 600 nm and a diameter of 500 nm, with five rows of holes on either side of the device, and four rows in the central region between the waveguides.

All devices use integrated grating couplers to couple light on and off the chip.

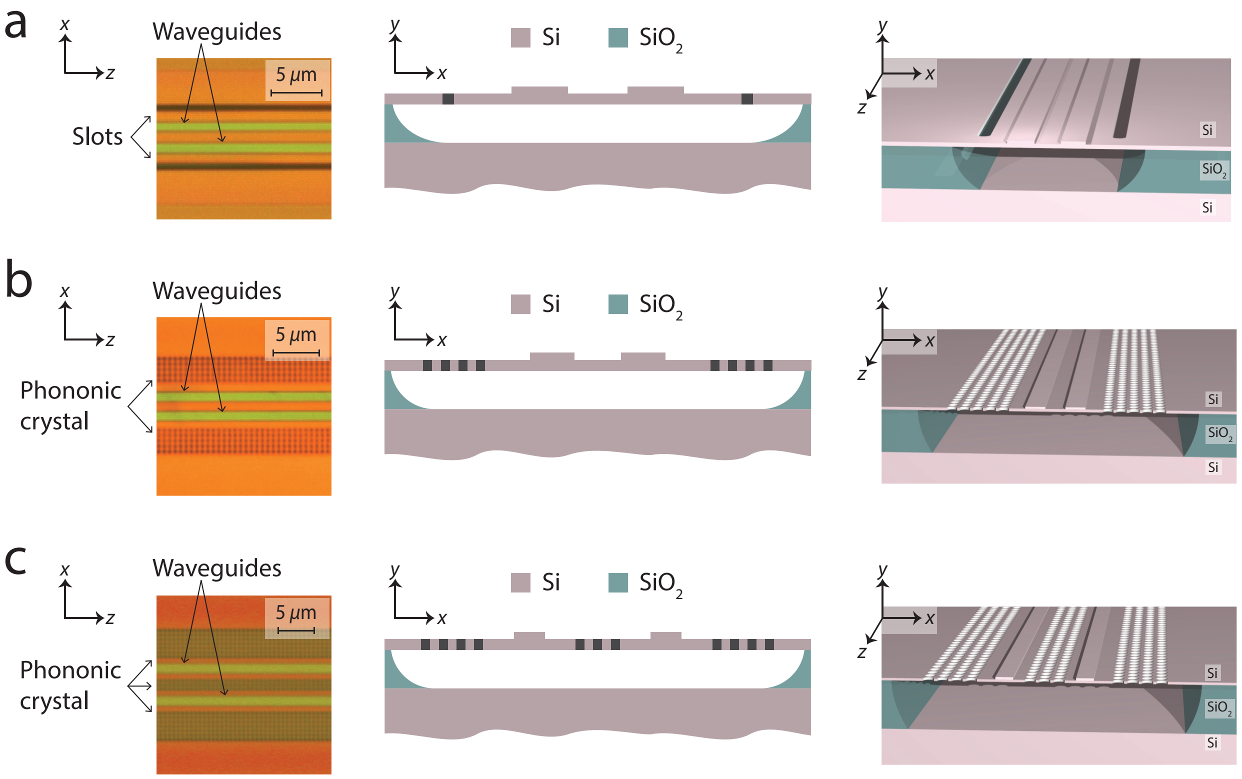

FIG. S8: **(a)** PPER device utilizing slots to confine the acoustic mode. **(b)** PPER device utilizing a phononic crystal structure to confine the acoustic mode. **(c)** Two-pole PPER device utilizing a phononic crystal structure to confine two coupled the acoustic modes. Left column: Micrograph of the fabricated devices. Middle column: Illustration of the device cross section. Right column: Artistic illustration of the device.

V. RF LINK PROPERTIES

Next, we use the results developed in Section II to calculate key RF link metrics and to explore potential performance as a function of system parameters. We return to Eq. (12), and calculate the average output RF power using $P_{\rm out}^{(\Omega)} = \langle I^2 \rangle R_{\rm out}|H|^2$, where $R_{\rm out}$ is the output impedance of the detector, and H is the photodiode circuit efficiency [23]. Expressing the input signal in terms of RF power, using $P_{\rm in}^{(\Omega)} = V_{\rm in}^2/(2R_{\rm in})$, where $R_{\rm in}$ is the intensity modulator input impedance, we have

$$P_{\rm out}^{(\Omega)} = \frac{1}{4} P_{\rm in}^{(\Omega)} \eta^2 R_{\rm out} |H|^2 R_{\rm in} \left(\frac{\pi}{V_\pi}\right)^2 \left(P^{(\rm B)} G_{\rm B} P^{(\rm E)} L\right)^2 |\xi(\Omega)|^2, \tag{19}$$

from which we can calculate the link gain $g = P_{\rm out}^{(\Omega)}/P_{\rm in}^{(\Omega)}$ in the filter pass-band (where $\xi(\Omega) \to 1$)

$$g = \frac{1}{4} \eta^2 R_{\rm out} |H|^2 R_{\rm in} \left(\frac{\pi}{V_\pi}\right)^2 \left(P^{(\rm B)} G_{\rm B} P^{(\rm E)} L\right)^2. \tag{20}$$

We can see that the link gain increases with higher optical powers, device length, and Brillouin gain, consistent with the measurements shown in Fig. 4 in the main text. Furthermore, a lower half-wave voltage of the intensity modulator at the link input (V_π) results in higher gain, as shown in the calculations presented in Fig. S9(a).

Next, we consider possible noise sources in the RF link, with a noise spectral density given by [23, 27]

$$N = \underbrace{(1+g)\, k_{\rm B} T}_{\text{Thermal noise}} + \underbrace{2qR_{\rm out}\, |H|^2\, I^{(\rm DC)}}_{\text{Shot noise}} + \underbrace{4\eta n_{\rm sp} \hbar\omega \, (g_{\rm EDFA} - 1)\, R_{\rm out}\, |H|^2\, I^{(\rm DC)}}_{\text{EDFA noise}}, \tag{21}$$

where the DC current $I^{(\rm DC)}$ was given in Eq. (13). The first term accounts for Johnson-Nyquist noise at the link input and output, proportional to the link gain, where $k_{\rm B}$ is the Boltzmann constant, and T is the temperature. The second term is shot noise, where q is the electron charge. The third term considers the noise from an optical erbium-doped fiber amplifier (EDFA), where we assume the main contribution is the beating of spontaneous emission with the signal [23], where $n_{\rm sp}$ is the spontaneous emission factor (of order unity), and $g_{\rm EDFA}$ is the amplifier gain. Fig. S9(b) shows the scaling of these noise sources with optical power and device length, for different cases of modulator half-wave voltage values. We can see that the noise added when using an EDFA is the dominant term and determines the noise floor of the microwave link.

We can calculate the noise factor (F) of the microwave-photonic link, a measure of the degradation of signal-to-noise ratio, given by

$$F = \frac{\mathrm{SNR}_{\rm in}}{\mathrm{SNR}_{\rm out}} = \frac{P_{\rm in}^{(\Omega)}/N_{\rm in}}{P_{\rm out}^{(\Omega)}/N_{\rm out}} = \frac{N_{\rm out}}{g N_{\rm in}}, \tag{22}$$

where we typically assume thermal noise at the link input (i.e., $N_{\rm in} = k_{\rm B} T = -174$ dBm/Hz). Using Eqs. (20) and (21) for the gain and output noise, we can calculate the noise figure of the link ($\mathrm{NF} = 10\log_{10}(F)$), as shown in Fig. S9(c) for the case of a link using an EDFA, and in Fig. S9(e) for a link without an EDFA. In both cases, we see the reduction in noise figure for stronger Brillouin interactions, consistent with our experimental results.

The main source of nonlinearity in the microwave-photonic link is the intensity modulator at the link input [24]. When operating the modulator at quadrature, the third-order nonlinearity is the dominant source of distortion [23]. Following the derivation in Ref. [24], we can calculate the third-order output intercept point (OIP_3)

$$\mathrm{OIP}_3 = 3\eta^2 R_{\rm out} |H|^2 \left(P^{(\rm B)} G_{\rm B} P^{(\rm E)} L\right)^2, \tag{23}$$

from which we can derive the third-order spur-free dynamic range, given by $\mathrm{SFDR}_3 = (\mathrm{OIP}_3/(N B_{\rm RF}))^{2/3}$, where $B_{\rm RF}$ is the RF bandwidth measured at the link output. Figs. S9(d) and S9(f) show the calculated dynamic range, with and without optical amplification, respectively. These results show an increase in the dynamic range with stronger Brillouin interactions, consistent with our experimental demonstration.

The calculations presented here show the potential of PPER-based notch filters to yield a link gain of 20 dB, a noise figure of ~20 dB, and a spur-free dynamic range >100 dB $\mathrm{Hz}^{2/3}$, as can be seen in Figs. S9(c) and S9(d). Furthermore, full integration of the filter on-chip could eliminate the need for optical amplification, which will reduce the noise floor (see Fig. S9(b)). The lower noise will enhance system performance, potentially improving the noise figure by 30 dB, and the dynamic range by 20 dB $\mathrm{Hz}^{2/3}$, as can be seen in Figs. S9(e) and S9(f).

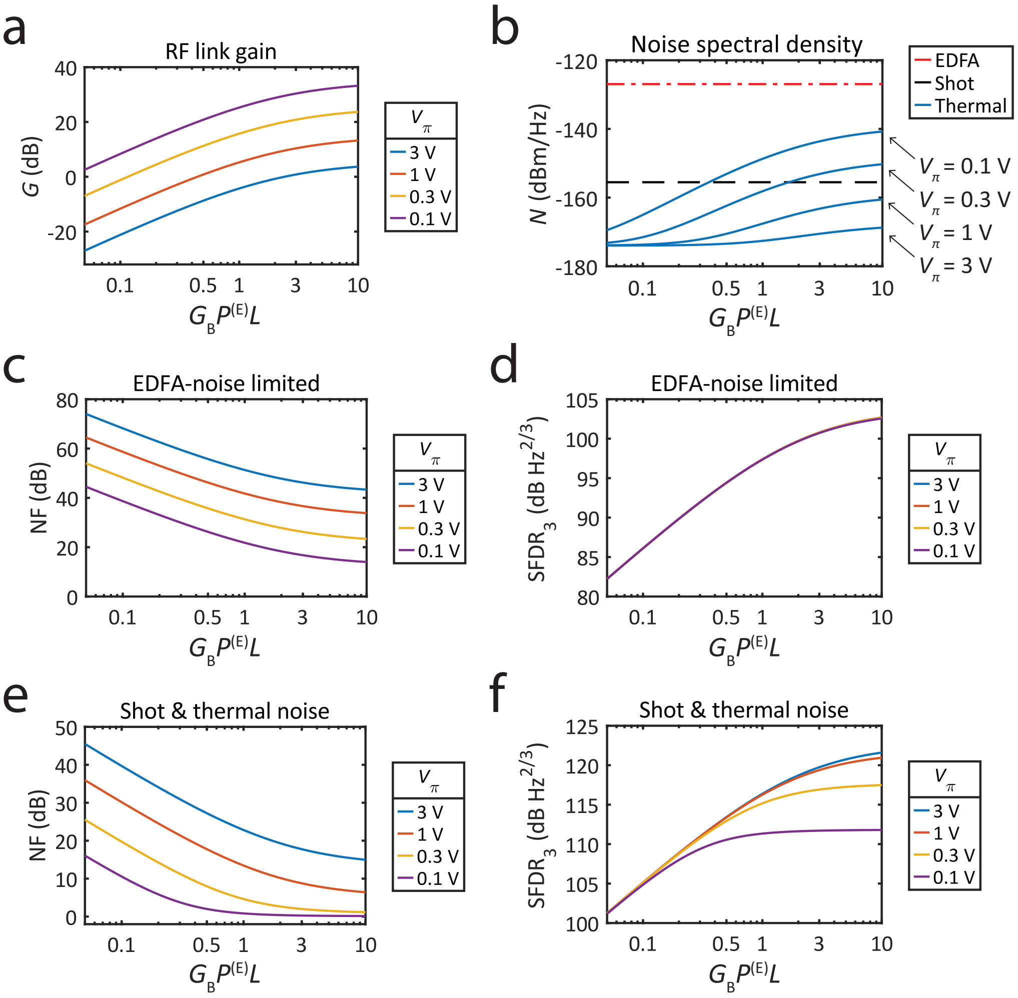

FIG. S9: Calculated RF link performance as a function of the acousto-optic interaction strength in the PPER device (given by the product of the optical power in the 'emit' waveguide ($P^{(E)}$), the active-region length (L), and the Brillouin gain (G_B)). The values used for calculations are typical to the device and link demonstrated in this work, assuming 100 mW of optical power on the detector. **(a)** RF link gain, for different values of modulator half-wave voltages. **(b)** Noise spectral density of the noise sources considered in Eq. (21). **(c)** Noise figure, assuming all three noise sources are present. **(d)** Spur-free dynamic range, assuming all three noise sources are present. **(e)** Noise figure, in the case of a system without an optical amplifier (EDFA). **(f)** Spur-free dynamic range, in the case of a system without an optical amplifier (EDFA).

VI. FREQUENCY RESPONSE MANIPULATION

Notch-frequency selection

The cancellation of the microwave signal at the notch frequency is a result of the coherent destructive interference between the signals from the two interferometer paths. To achieve strong suppression, both the phase and amplitude of the signals have to be balanced (see Fig. S3). In the case of a PPER device supporting multiple acoustic modes, the frequency at which the cancellation occurs can be selected by matching the power and time-delay in the reference arm of the interferometer to that of different signals from the PPER output, which correspond to different acoustic modes. Multiple acoustic modes can be present in the same device or could be a result of multiple PPER segments designed to have different resonant frequencies (see Section VIII).

As an example, we consider a single-pole PPER with two acoustic resonances at frequencies $\Omega_0^{(1)}$ and $\Omega_0^{(2)}$, with Brillouin gains $G_B^{(1)}$ and $G_B^{(2)}$, respectively. Following our derivation in the previous section, the output RF power can be written as

$$P_{\text{out}}^{(\Omega)} = P_{\text{in}}^{(\Omega)} \eta^2 R_{\text{out}} \left|H\right|^2 R_{\text{in}} \left(\frac{\pi}{V_\pi}\right)^2 \left| P^{(A)} - \frac{1}{2} P^{(B)} G_B^{(1)} P^{(E)} L \frac{\Gamma}{2} \chi^{(1)}(\Omega) - \frac{1}{2} P^{(B)} G_B^{(2)} P^{(E)} L \frac{\Gamma}{2} \chi^{(2)}(\Omega) \right|^2, \tag{24}$$

where we have assumed the path lengths are matched, such that $\Delta\tau = 0$. We can use the difference in Brillouin gain to select the frequency at which strong signal suppression will occur. If we set the power $P^{(A)}$ to balance the first resonance, i.e., $P^{(A)} = P^{(B)} G_B^{(1)} P^{(E)} L/2$, we will get cancellation of the signal at $\Omega_0^{(1)}$, while at frequency $\Omega_0^{(2)}$ we will have

$$P_{\text{out}}^{(\Omega)}\left(\Omega_0^{(2)}\right) = \frac{1}{4} P_{\text{in}}^{(\Omega)} \eta^2 R_{\text{out}} \left|H\right|^2 R_{\text{in}} \left(\frac{\pi}{V_\pi}\right)^2 \left(P^{(B)} G_B^{(1)} P^{(E)} L\right)^2 \left|1 - \frac{G_B^{(2)}}{G_B^{(1)}}\right|^2. \tag{25}$$

Here, we have assumed that the two acoustic modes are well separated, such that $\chi^{(1)}(\Omega_0^{(2)}) = \chi^{(2)}(\Omega_0^{(1)}) = 0$. Alternatively, if we set the optical power $P^{(A)}$ to match the second resonance, such that $P^{(A)} = P^{(B)} G_B^{(2)} P^{(E)} L/2$, we will get perfect cancellation at frequency $\Omega_0^{(2)}$, while at frequency $\Omega_0^{(1)}$ the power is given by

$$P_{\text{out}}^{(\Omega)}\left(\Omega_0^{(1)}\right) = \frac{1}{4} P_{\text{in}}^{(\Omega)} \eta^2 R_{\text{out}} |H|^2 R_{\text{in}} \left(\frac{\pi}{V_\pi}\right)^2 \left(P^{(B)} G_B^{(2)} P^{(E)} L\right)^2 \left|1 - \frac{G_B^{(1)}}{G_B^{(2)}}\right|^2. \tag{26}$$

We assume that the Brillouin gain of two modes differs by 3 dB, i.e., $G_B^{(1)} = 2G_B^{(2)}$, and that they are separated in frequency by 1 GHz. When the signal is fully suppressed at frequency $\Omega_0^{(1)} = 3.5$ GHz, there will be a 6 dB dip at

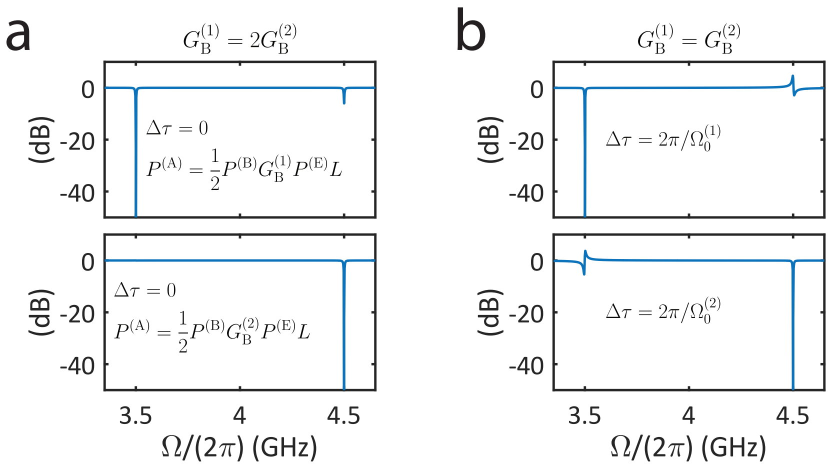

FIG. S10: **(a)** The notch frequency can be switched between two resonances with different Brillouin gains by matching the power to each resonance, assuming no time-delay between the two interferometer paths. **(b)** In the case of two resonances with identical gain, the phase induced by the time-delay between the two interferometer paths can be used to select the notch frequency.

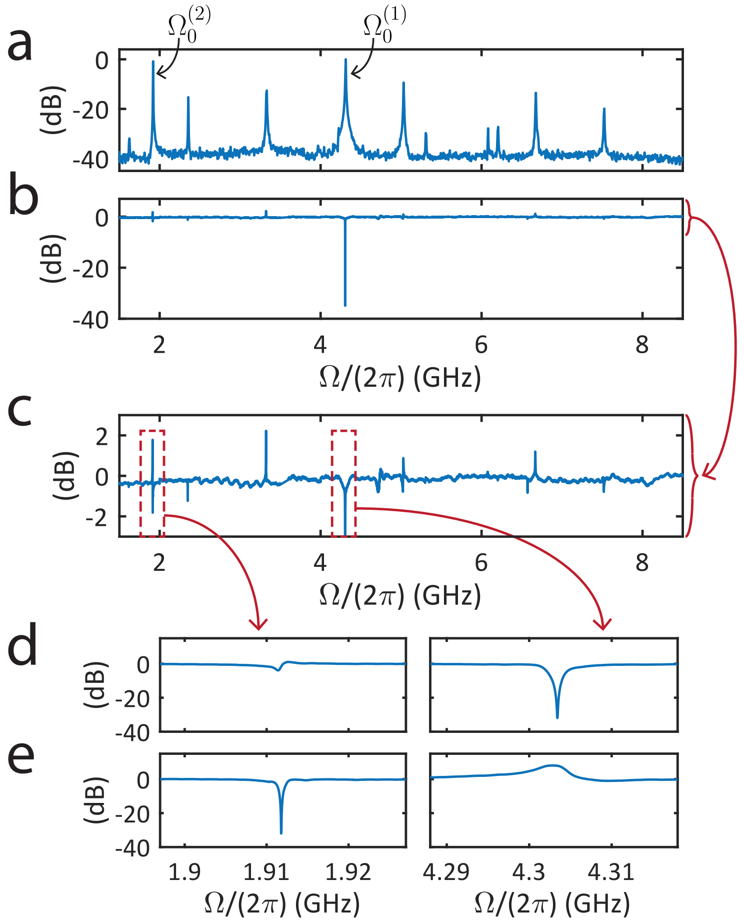

FIG. S11: **(a)** The measured frequency response of a PPER device reveals multiple acoustic modes. **(b)** The corresponding notch filter response, when the interferometer is set to achieve cancellation of the signal corresponding to the mode at frequency $\Omega_0 = 4.3$ GHz. **(c)** A magnified view shows spurs at frequencies corresponding to other acoustic modes. **(d)** Magnified view, showing the distortion around frequency $\Omega_0 = 1.91$ GHz and $\sim$ 40 dB of cancellation at frequency $\Omega_0 = 4.3$ GHz. **(e)** By tuning the interferomenter using a variable optical amplifier (VOA), we shift the notch filter to achieve signal cancellation at frequency $\Omega_0 = 1.91$ GHz.

$\Omega_0^{(2)} = 4.5$ GHz, as seen in the calculation shown in Fig. S10(a). In this example, when the amplitude is matched to cancel the signal at $\Omega_0^{(2)}$, there will be no ripple in the frequency response at $\Omega_0^{(1)}$.

We demonstrate the selection of the notch frequency experimentally, using a PPER device supporting multiple high-Q acoustic resonances, seen in Fig. S11(a). By balancing the power of the resonance at frequency $\Omega_0^{(1)} = 4.3$ GHz, we achieve a notch filter with $\sim$40 dB rejection (see Fig. S11(b)), while the other resonances produce a ripple of a few dB, as shown in the magnified view in Figs. S11(c) and S11(d). By tuning the power $P^{(\mathrm{A})}$, matching it to the resonance at frequency $\Omega_0^{(2)} = 1.91$ GHz, we shift the $\sim$ 40 dB notch response to $\Omega_0^{(2)}$, leaving a distortion of 8 dB at $\Omega_0^{(1)}$, seen in Fig. S11(e). While this demonstration utilized acoustic modes within the same PPER device that have different Brillouin gain, a similar calculation can be carried out for the case of multiple PPER segments. In this case, the active segments can be designed to have different active lengths L, resulting in an amplitude difference between their corresponding signals, which can be similarly used for notch frequency selection.

An alternative strategy for notch-frequency selection is to utilize the different phase shifts accumulated by different frequency components for a given time delay. We consider a system with two well-separated acoustic modes, which we now assume to have equal Brillouin gain, and match the optical powers of the two interferometer paths, such that

$$P_{\mathrm{out}}^{(\Omega)}\left(\Omega_0^{(1)}\right) = \frac{1}{4} P_{\mathrm{in}}^{(\Omega)} \eta^2 R_{\mathrm{out}} |H|^2 R_{\mathrm{in}} \left(\frac{\pi}{V_\pi}\right)^2 \left(P^{(\mathrm{B})} G_{\mathrm{B}} P^{(\mathrm{E})} L\right)^2 \left|1 - \left(\frac{\Gamma}{2}\chi^{(1)}(\Omega) + \frac{\Gamma}{2}\chi^{(2)}(\Omega)\right) e^{-i\Omega\Delta\tau}\right|^2. \tag{27}$$

Here, we can use the time-delay $\Delta\tau$ to select the frequency at which perfect signal cancellation occurs. By setting $\Delta\tau = 2\pi m/\Omega_0^{(1)}$ (for integer m), strong signal suppression will occur at frequency $\Omega_0^{(1)}$. By changing the time delay,

such that $\Delta\tau = 2\pi m/\Omega_0^{(2)}$, we can shift the notch frequency to $\Omega_0^{(2)}$. This is demonstrated numerically in Fig. S10(b), where we have chosen the value $m = 1$, showing the shifting of the notch filter between two frequencies, with a distortion of a few dB at the unsuppressed resonance.

Inverting the frequency response

As we have seen in the previous sections, the frequency response of the notch filter is set by the phase relation between the two interferometer arms. When the signals are exactly out of phase, we achieve a notch filter response, assuming both signals have equal powers. If, however, the signals add in phase, by setting the time delay such that $\Delta\tau = \pi/\Omega_0 + 2\pi m$ (for integer m), the resulting frequency response will show a peak of 6 dB at the resonance frequency (see Fig. S3(a)). The ability to manipulate the frequency response using a phase shift can be utilized for switching applications, where the notch filter can be effectively turned on and off through control of a time delay.

The change in the relative phase between the two signals can also be controlled through the demodulation scheme we are implementing at the PPER output. The demodulation we have used in our demonstration utilizes optical filtering of the light exiting the 'receive' waveguide of the PPER, such that only the optical carrier (at frequency ω_2) and the first higher sideband (indices $n = 0, 1$ from Eq. (4)) are transmitted. However, if we use the optical filter to select the carrier and the first lower sideband (indices $n = -1, 0$), and repeat the steps of the derivation from Section II, the photocurrent at the detector will be given by

$$I^{(\Omega)}(t) = -\eta\left(\frac{\pi V_{\text{in}}}{V_\pi}\right)\text{Im}\left[e^{-i\Omega t}\left(P^{(\text{A})} + \frac{1}{2}P^{(\text{B})}G_{\text{B}}P^{(\text{E})}L\frac{\Gamma}{2}\left|\chi(\Omega)\right|e^{-i(\Omega\Delta\tau-\phi)}\right)\right]. \tag{28}$$

When the power $P^{(\text{A})}$ is set to the value needed for notch filtering and the time-delays of the two optical paths are matched ($\Delta\tau = 0$), the output RF power will be

$$P_{\text{out}}^{(\Omega)} = \frac{1}{4}P_{\text{in}}^{(\Omega)}\eta^2 R_{\text{out}}|H|^2 R_{\text{in}}\left(\frac{\pi}{V_\pi}\right)^2\left(P^{(\text{B})}G_{\text{B}}P^{(\text{E})}L\right)^2\left|1 + \frac{\Gamma}{2}\chi(\Omega)\right|^2. \tag{29}$$

On resonance ($\Omega = \Omega_0$), this yields a combined power that is four times larger than the power off-resonance, since $(\Gamma/2)\chi(\Omega_0) = 1$ and $\chi(\Omega \neq \Omega_0) \to 0$). Hence, by switching the sideband selected in the PPER demodulation, the notch filter response can be transformed into a peak of 6 dB in transmission. We demonstrate this experimentally, switching between a ∼40 dB dip and a 6 dB peak, as seen in Fig. S12.

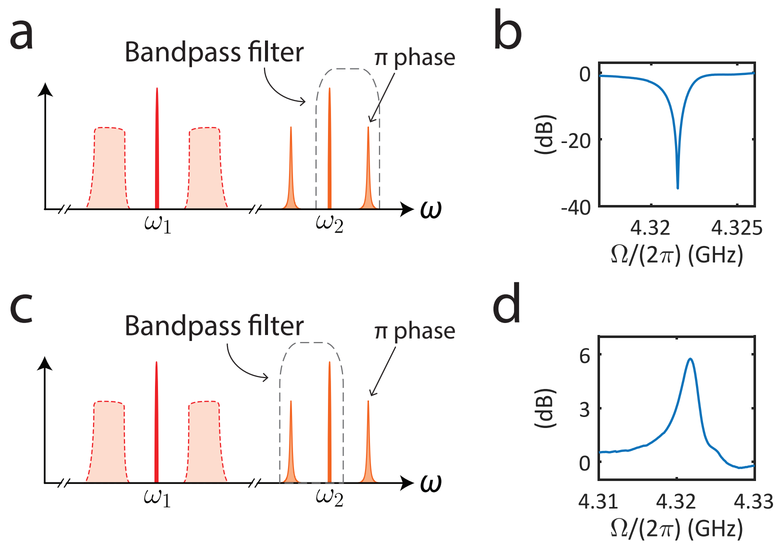

FIG. S12: **(a)** When the beat-note produced by the intensity modulator is out of phase with the demodulated PPER output (after passing through a band-pass filter), we achieve cancellation at the Brillouin frequency. **(b)** An example of a measured frequency response showing this cancellation. **(c)** By tuning the bandpass filter, the demodulated signal can be in-phase with the one produced by the intensity modulator. **(d)** The response of the filter after tuning the band-pass filter, showing the addition of the power at the Brillouin frequency, yielding a 6 dB peak.

VII. ALTERNATIVE NOTCH-FILTERING IMPLEMENTATIONS

Frequency-neutral tunable filter

The tunable filtering scheme demonstrated in this work, illustrated in Fig. 3(a) of the main text, results in a frequency shift of the RF signal at the filter output compared to the input RF signal. This frequency conversion can be advantageous in certain systems where frequency down/up conversion are necessary. However, in some applications, where the filter is part of a larger microwave-photonic system, complementary strategies could be needed in which we can achieve a frequency-neutral operation.

An example of a possible implementation of a frequency-neutral tunable PPER-based narrowband microwave-photonic filter is illustrated schematically in Fig. S13. Here, the RF input signal is encoded onto an optical carrier with optical frequency ω_1 using a phase modulator. This light is combined with a second optical tone, which serves as a local oscillator, at an optical frequency ω_{LO}. The combined light is split into two paths: one path bypassing the PPER device and the other path directed into the 'emit' waveguide of the PPER device. To achieve filtering at microwave frequency Ω_{filt}, we set the local oscillator such that $\omega_{LO} = \omega_1 + \Omega_{filt} + \Omega_0$, where Ω_0 is the Brillouin frequency of the PPER device (see bottom left inset in Fig. S13). The intensity beat-note at the Brillouin frequency in the 'emit' waveguide will drive phonons in the device, resulting in phase-modulation sidebands on an optical carrier propagating in the 'receive' waveguide of the device. The input RF signal is also used to intensity-modulate a second optical tone at frequency ω_0, which is combined with the output light from the PPER and the local oscillator (see bottom right inset in Fig. S13). At the detector, there will be two beat-notes at frequency Ω_{filt}; one is the result of the intensity modulated light, and the other is the local oscillator beating with the Brillouin-induced sideband, whose amplitude and phase can be set to achieve signal cancellation. In this way, the output RF signal follows the input RF frequency, with a tunable narrowband notch frequency response at frequency $\Omega_{filt} = \omega_{LO} - \omega_1 - \Omega_0$, which can be shifted by tuning the frequency of the local oscillator (ω_{LO}).

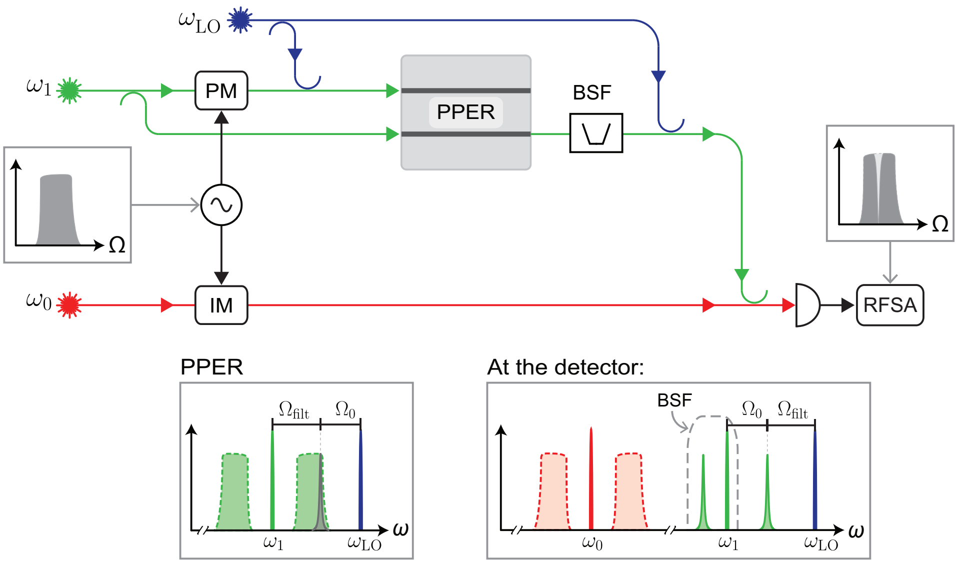

FIG. S13: Schematic illustration of a frequency-neutral tunable PPER-based narrowband microwave-photonic filter. PM: phase modulator, IM: intensity modulator, BSF: optical bandstop filter, RFSA: RF spectrum analyzer.

Using two photo-detectors

In the notch-filtering scheme we have presented, the signals from the two interferometer paths were combined in the optical domain before detection. However, there was no optical interference in this scheme, as the wavelengths of the two fields were well separated, and the interference was achieved in the microwave domain (i.e., the photocurrent in the detector). A possible variation to the PPER-based notch filter we have presented is to use two photodetectors, each detecting the light from one of the interferometer paths. By combining the photocurrents from the two detectors out of phase, we can achieve signal cancellation, as is typically implemented in balanced detection schemes. One such scheme is presented in Fig. S14(a), where each path of the optical interferometer is detected separately, and the signals from the two photodetectors are combined using an RF hybrid (i.e., RF directional coupler) to achieve the desired notch filter response.

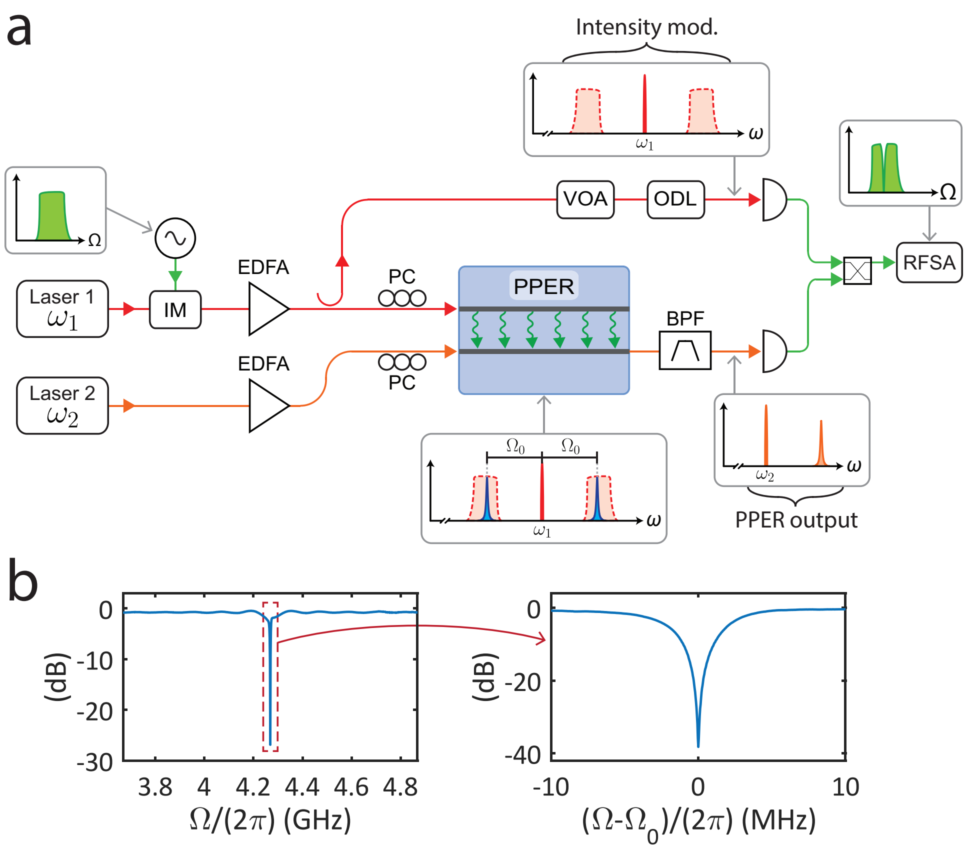

FIG. S14: **(a)** Schematic illustration of the experimental setup used to demonstrate PPER-based notch filtering using two photo-detectors. IM: intensity modulator, VOA: variable optical attenuator, ODL: optical delay line, BPF: optical bandpass filter, RFSA: RF spectrum analyzer. EDFA: erbium-doped fiber amplifier, PC: polarization controller. **(b)** Measured frequency response of the notch filter. Right: Magnified view around the filter notch, showing 40 dB of signal suppression.

Analyzing this scenario, the field impinging on the first photodetector is the output of the intensity modulator, which was described in Eq. (1). The photocurrent produced by this detector at the RF input frequency (Ω) is given by

$$I_{\mathrm{A}}^{(\Omega)} = -2\eta^{(\mathrm{A})}\widetilde{P}^{(\mathrm{A})}J_1\left(\frac{\pi V_{\mathrm{in}}}{V_\pi}\right)\sin\Omega t, \tag{30}$$

where $\eta^{(\mathrm{A})}$ is the responsivity of the detector. The photocurrent produced at the second detector is a result of the demodulated PPER output, which was described in Eq. (5), and calculating the photocurrent at frequency Ω gives us

$$I_{\mathrm{B}}^{(\Omega)} = 2\eta^{(\mathrm{B})}\widetilde{P}^{(\mathrm{B})}J_0\left(\beta_{\mathrm{in}}\right)J_1\left(\beta_{\mathrm{in}}\right)\sin\left(\Omega t-\phi\right), \tag{31}$$

where $\eta^{(\mathrm{B})}$ is the responsivity of the second detector. Combining the currents from Eqs. (30) and (31) yields

$$I^{(\Omega)}(t) = 2\left[\eta^{(\mathrm{B})}P^{(\mathrm{B})}J_0\left(\beta_{\mathrm{in}}\right)J_1\left(\beta_{\mathrm{in}}\right)\sin\left(\Omega\left(t-\tau^{(\mathrm{B})}\right)-\phi\right)-\eta^{(\mathrm{A})}P^{(\mathrm{A})}J_1\left(\frac{\pi V_{\mathrm{in}}}{V_\pi}\right)\sin\left(\Omega\left(t-\tau^{(\mathrm{A})}\right)\right)\right], \tag{32}$$

which has a similar form to Eq. (7) from the scheme discussed in Section II. The time delays $\tau^{(\mathrm{A})}$ and $\tau^{(\mathrm{B})}$ correspond to the time-delays of the two signals, which can be adjusted in the optical or in the RF domain. The relation between the powers $\{\widetilde{P}^{(\mathrm{A})},\widetilde{P}^{(\mathrm{B})}\}$ and $\{P^{(\mathrm{A})},P^{(\mathrm{B})}\}$ is determined by the ratio in which the currents are combined. Analyzing the performance of this scheme is equivalent to that presented in the previous section, where a possible difference in the responsivity of the two detectors needs to be taken into account. An experimental demonstration of filtering using this scheme is presented in Fig. S14(b), showing the measurement of a PPER-based notch filter with 40 dB of signal suppression at the notch frequency.

Signal interference in the optical domain

Alternatively, signal cancellation can be achieved directly in the optical domain, rather than through RF signal interference. This filtering scheme is shown schematically in Fig. S15(a), illustrating the cancellation of the optical sidebands at the notch frequency before detection. By using the same laser source for both the 'emit' and 'receive' waveguides of the PPER, all signals are optically coherent, and the optical sidebands carrying the RF information directly interfere in the optical domain, resulting in the cancellation of the optical signal at an optical frequency spaced by the Brillouin frequency from the carrier. After detection, this corresponds to suppression of the RF signal at the Brillouin frequency. In this case, the interference occurs in the optical domain, and implementing such a microwave-photonic filter requires a higher level of stabilization, which could be achieved by a fully integrated device [15].

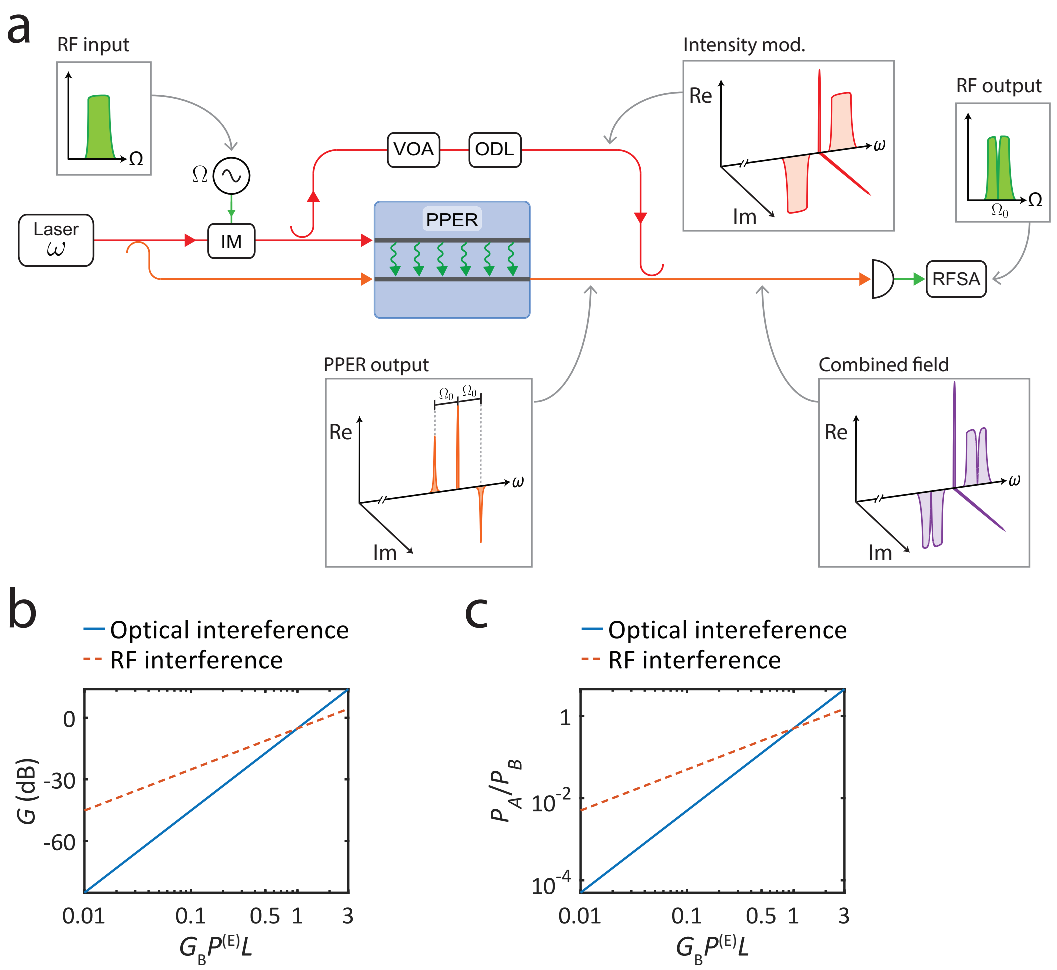

FIG. S15: **(a)** PPER-based notch filtering scheme using optical interference. IM: intensity modulator, VOA: variable optical attenuator, ODL: optical delay line, RFSA: RF spectrum analyzer. **(b)** Calculated RF link gain and **(c)** optical power needed to achieve signal cancellation as a function of the Brillouin interaction strength (solid blue line). The equivalent calculation for the RF-interference notch-filtering scheme (see Section II) is shown for reference (dashed red line).

Using the expression for the combined field at the detector from Eq. (6), and assuming both fields have the same optical carrier frequency ω, we have

$$E(t) = \sqrt{\frac{P^{(\mathrm{A})}}{2}} e^{-i\omega t}\left(i + \sum_n J_n\left(\frac{\pi V_{\mathrm{in}}}{V_\pi}\right) e^{-in\Omega t}\right) + i\sqrt{P^{(\mathrm{B})}}\; e^{-i\omega(t+\Delta\tau)} \sum_n i^{-n} J_{-n}\left(\beta_{\mathrm{in}}\right) e^{-in(\Omega(t+\Delta\tau)-\phi)}, \tag{33}$$

where we have defined the time delay between the two signals as $\Delta\tau$. Next, we set the time delay such that $\omega\Delta\tau = \pi/2 + 2\pi m$ (for integer m), resulting in

$$E(t) = \sqrt{\frac{P^{(\mathrm{A})}}{2}} e^{-i\omega t}\left(i + \sum_n J_n\left(\frac{\pi V_{\mathrm{in}}}{V_\pi}\right) e^{-in\Omega t}\right) + \sqrt{P^{(\mathrm{B})}}\; e^{-i\omega t} \sum_n J_{-n}\left(\beta_{\mathrm{in}}\right) e^{-in(\Omega(t+\Delta\tau)-\phi+\pi/2)}. \tag{34}$$

For small RF signals (i.e., $V_{\mathrm{in}} \ll V_\pi$), we can expand the Bessel functions to first order ($J_0(x) \approx 1, J_{\pm1}(x) \approx \pm x/2$), neglecting higher order terms. Substituting $\beta_{\mathrm{in}} \approx G_{\mathrm{B}} P^{(\mathrm{E})} L(\pi V_{\mathrm{in}}/V_\pi)|\chi(\Omega)|\Gamma/4$ (see Eq. (3)), we have

$$E(t) = e^{-i\omega t}\left[\left(i\sqrt{\frac{P^{(\mathrm{A})}}{2}} + \sqrt{\frac{P^{(\mathrm{A})}}{2}} + \sqrt{P^{(\mathrm{B})}}\right) + i\left(\frac{\pi V_{\mathrm{in}}}{V_\pi}\right) \mathrm{Im}\left[e^{-i\Omega t}\left(\sqrt{\frac{P^{(\mathrm{A})}}{2}} - \frac{1}{2}\sqrt{P^{(\mathrm{B})}} G_{\mathrm{B}} P^{(\mathrm{E})} L \frac{\Gamma}{2} \left|\chi(\Omega)\right| e^{-i(\Omega\Delta\tau - \phi + \pi/2)}\right)\right]\right]. \tag{35}$$

To achieve cancellation at the Brillouin frequency, we set the optical power $P^{(\mathrm{A})}$ such that $P^{(\mathrm{A})} = P^{(\mathrm{B})}(G_{\mathrm{B}} P^{(\mathrm{E})} L)^2/2$, yielding

$$E(t) = \sqrt{P^{(\mathrm{B})}}\; e^{-i\omega t}\left[\left(1 + \frac{1}{2} G_{\mathrm{B}} P^{(\mathrm{E})} L + \frac{i}{2} G_{\mathrm{B}} P^{(\mathrm{E})} L\right) + i\left(\frac{\pi V_{\mathrm{in}}}{V_\pi}\right)\left(\frac{1}{2} G_{\mathrm{B}} P^{(\mathrm{E})} L\right) \mathrm{Im}\left[e^{-i\Omega t}\left(1 - \frac{\Gamma}{2}|\chi(\Omega)| e^{-i(\Omega\Delta\tau - \phi + \pi/2)}\right)\right]\right]. \tag{36}$$

Calculating the photo current at the detector ($I = \eta|E|^2$), keeping the terms oscillating at frequency Ω, we have

$$I^{(\Omega)}(t) = 2\eta\left(\frac{\pi V_{\mathrm{in}}}{V_\pi}\right) P^{(\mathrm{B})}\left(\frac{1}{2} G_{\mathrm{B}} P^{(\mathrm{E})} L\right)^2 \mathrm{Im}\left[e^{-i\Omega t}\left(1 - \frac{\Gamma}{2}|\chi(\Omega)| e^{-i(\Omega\Delta\tau - \phi + \pi/2)}\right)\right]. \tag{37}$$

Assuming a single-pole PPER device, such that at the Brillouin frequency we have $\phi(\Omega_0) = 0$ (see Eq. (10)), we set the time-delay $\Omega_0\Delta\tau = -\pi/2 + 2\pi m$ (for integer m), giving us

$$I^{(\Omega)}(t) = 2\eta\left(\frac{\pi V_{\mathrm{in}}}{V_\pi}\right) P^{(\mathrm{B})}\left(\frac{1}{2} G_{\mathrm{B}} P^{(\mathrm{E})} L\right)^2 \mathrm{Im}\left[e^{-i\Omega t}\left(1 - \frac{\Gamma}{2}\chi(\Omega) e^{-i\left[(\pi/2)(1-\Omega/\Omega_0) + 2\pi m(\Omega/\Omega_0)\right]}\right)\right], \tag{38}$$

which has a similar form to Eq. (12). A similar analysis can be performed for a two-pole PPER response, as was discussed in Section III.

We can calculate the RF power ($P_{\mathrm{out}}^{(\Omega)} = \langle I^2\rangle R_{\mathrm{out}}|H|^2$), yielding

$$P_{\mathrm{out}}^{(\Omega)} = \frac{1}{4} P_{\mathrm{in}}^{(\Omega)} \eta^2 R_{\mathrm{out}}|H|^2 R_{\mathrm{in}}\left(\frac{\pi}{V_\pi}\right)^2 P^{(\mathrm{B})^2}\left(G_{\mathrm{B}} P^{(\mathrm{E})} L\right)^4 \left|1 - \frac{\Gamma}{2}\chi(\Omega) e^{-i\left[(\pi/2)(1-\Omega/\Omega_0)\right]}\right|^2, \tag{39}$$

where we have expressed the input RF signal in terms of power ($P_{\mathrm{in}}^{(\Omega)} = V_{\mathrm{in}}^2/(2R_{\mathrm{in}})$). The RF link gain in the filter pass-band (i.e., $\chi(\Omega) \to 0$) is given by

$$g = \frac{1}{4}\eta^2 R_{\mathrm{out}}|H|^2 R_{\mathrm{in}}\left(\frac{\pi}{V_\pi}\right)^2 P^{(\mathrm{B})^2}\left(G_{\mathrm{B}} P^{(\mathrm{E})} L\right)^4. \tag{40}$$

We see that in this case, the RF link gain scales with the fourth power of the Brillouin interaction strength ($G_{\mathrm{B}} P^{(\mathrm{E})} L$), rather than the quadratic scaling we saw earlier. Fig. S15(b) shows the calculated RF link gain as

a function of $G_B P^{(E)} L$ (Brillouin gain, optical power in the 'emit' waveguide, and device length, respectively), and compares it to that obtained for the RF-interference notch-filtering scheme discussed in Section II. Fig. S15(c) presents the required optical power $P^{(A)}$ needed to achieve signal cancellation at the notch frequency. We can calculate the DC current when the power $P^{(A)}$ is set for the notch filtering operation

$$I^{(DC)} = \eta\left(P^{(A)} + P^{(B)} + \sqrt{2P^{(A)}P^{(B)}}\right), \tag{41}$$

from which we can derive the noise at the microwave-photonic link output using Eq. (21), as well as the noise figure and dynamic range (Eqs. (22) and (23)), seen in Fig. S16. We can see enhanced performance when increasing the Brillouin interaction strength, showing a higher gain, lower noise figure, and a larger dynamic range.

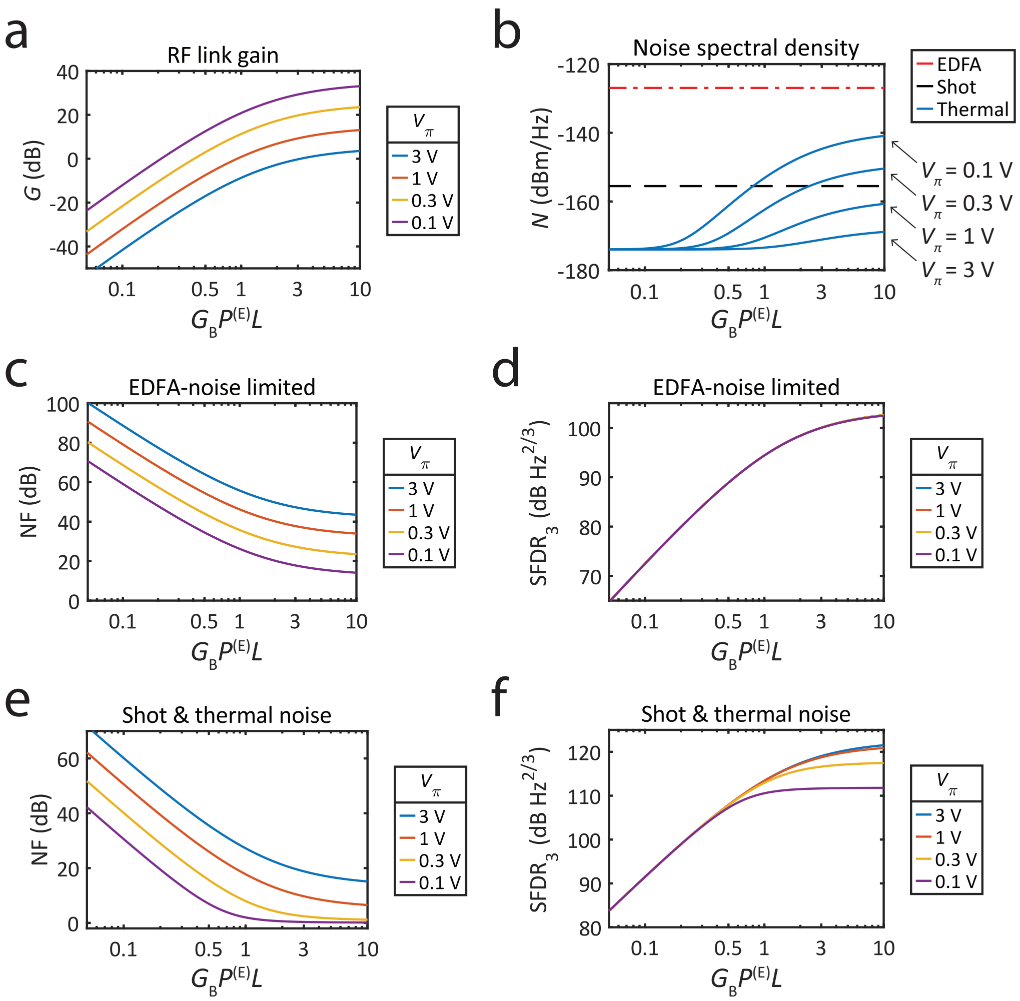

FIG. S16: Calculated RF link performance for the optical interference scheme (see Fig. S15) as a function of the acousto-optic interaction strength in the PPER device ($G_B P^{(E)} L$). The values used for calculations are typical to the device and link demonstrated in this work, assuming 100 mW of optical power on the detector. **(a)** RF link gain, for different values of modulator half-wave voltages. **(b)** Noise spectral density of the noise sources considered in Eq. (21). **(c)** Noise figure, assuming all three noise sources are present. **(d)** Spur-free dynamic range, assuming all three noise sources are present. **(e)** Noise figure, in the case of a system without an optical amplifier (EDFA). **(f)** Spur-free dynamic range, in the case of a system without an optical amplifier (EDFA).

VIII. FILTER ARRAYS

The unique properties of the PPER-based filtering scheme enable the design systems consisting of cascaded filtering sections, each processing a different frequency of the input signal. As an example, Fig. S17 shows a scheme where three PPER sections, each with a different Brillouin frequency, are cascaded in series. The Brillouin frequency of each section can be determined by the device geometry [26], and the low-loss waveguide design of the PPER device yields negligible signal degradation between the sections, which can all be integrated onto a single chip [24, 28]. The interference of the cascaded filters with the output of the intensity modulator will result in the cancellation of the RF signal at the Brillouin frequencies of all the sections. Variation in the Brillouin gain (G_B) between the different segments could be compensated through the length and the optical power in the 'emit' waveguide of each section. The multiple-notch frequency response can be tuned using the scheme presented in Fig. 3(a) of the main text. However, in this scheme, the spacing between the notch frequencies is fixed. Alternatively, through localized heating or by inducing strain to the separate PPER segments, the Brillouin frequency could be shifted, tuning the notch frequency.

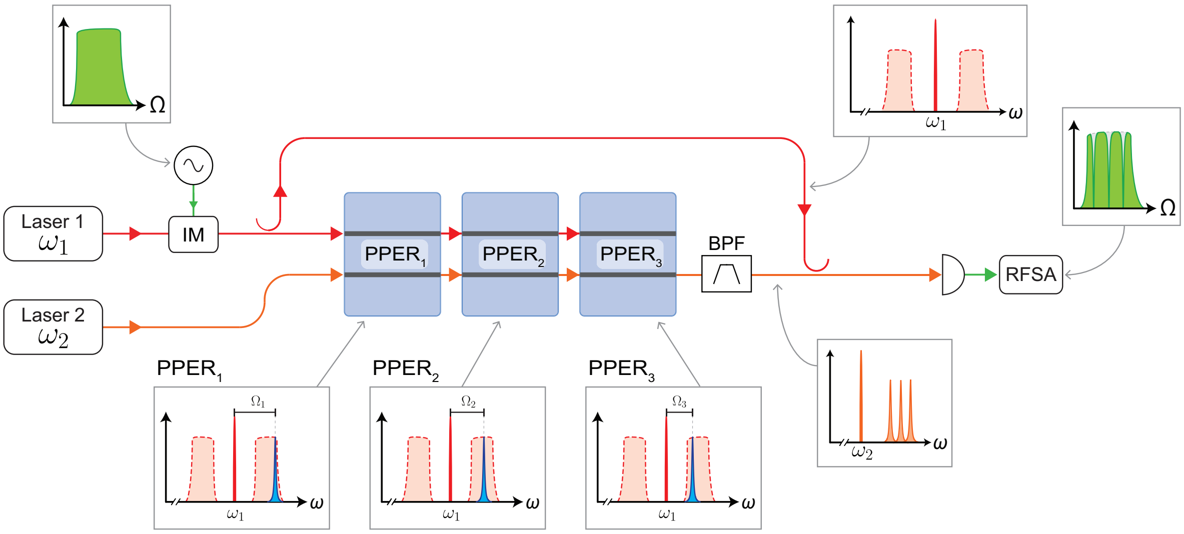

FIG. S17: Schematic illustration of three cascaded PPER filters used to produce three notch frequencies. In this scheme, the geometry of each PPER segment is designed to have a different Brillouin frequency. IM: intensity modulator, BPF: optical bandpass filter, RFSA: RF spectrum analyzer.

Another possible notch filtering scheme consisting of multiple PPER devices is illustrated in Fig. S18. Here, the PPER devices are set in parallel, and in each device, an optical local oscillator is used to select the spectral region that is being filtered. By combining the demodulated outputs of all the PPER devices, along with the output of an intensity modulator, multiple notch frequencies can be obtained, each separately tunable using the different local oscillators. While such a system requires more components compared with other schemes presented here, all of the necessary elements, namely modulators, filters, and optical directional couplers, can be integrated on-chip and are compatible with processes in existing silicon-photonic foundries.

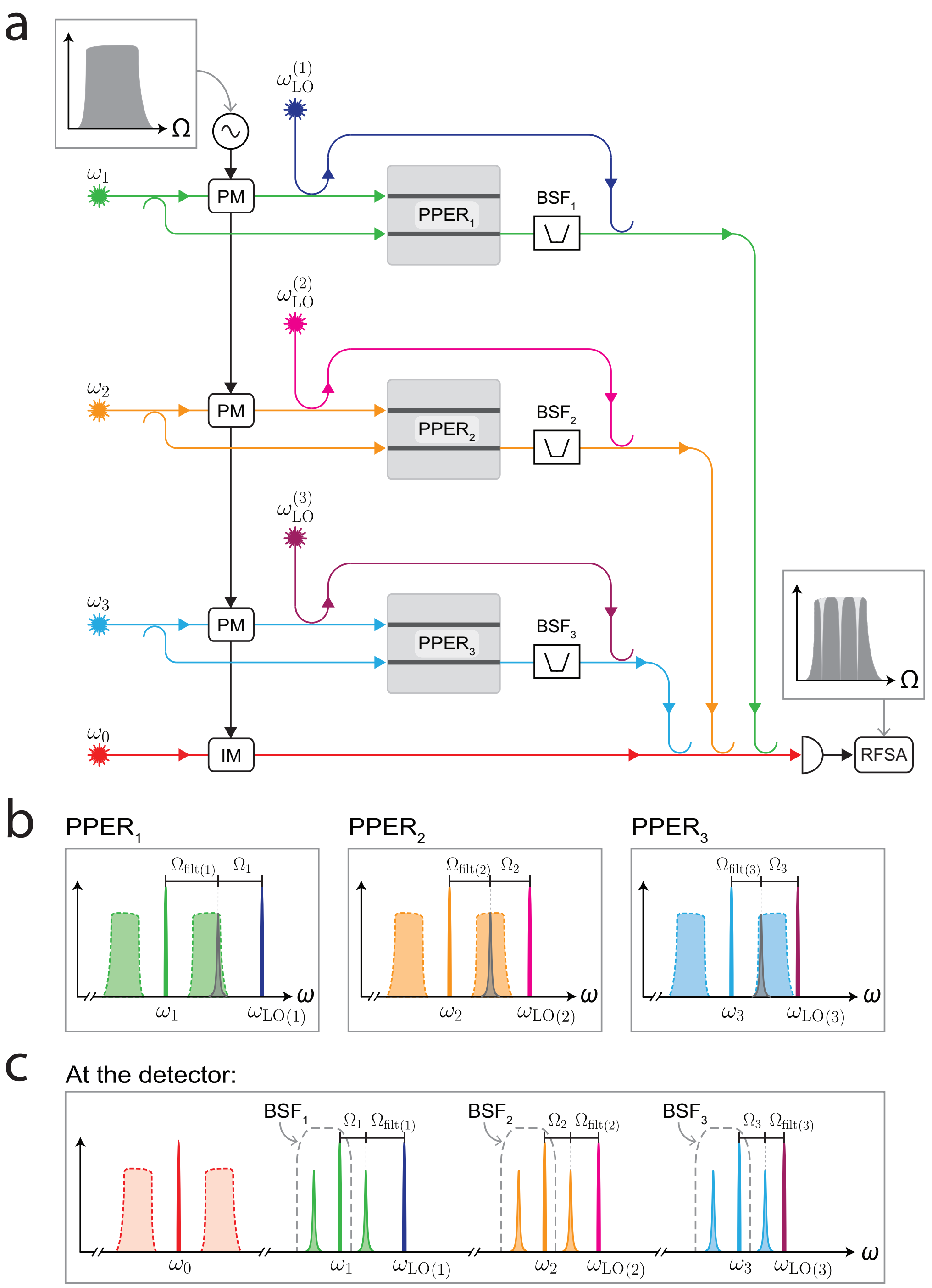

FIG. S18: **(a)** Schematic illustration of three PPER-based filters used to achieve independently tunable notch frequencies. PM: phase modulator, IM: intensity modulator, BSF: optical bandstop filter, RFSA: RF spectrum analyzer. **(b)** In each of the PPER devices, an optical local oscillator selects the spectral band that is transduced in the device. **(c)** At the detector, the signals from the different segments are combined, and their interference with the output of an intensity modulator results in the multiple-notch frequency response.

[1] Morrison, B. *et al.* Tunable microwave photonic notch filter using on-chip stimulated Brillouin scattering. *Opt. Commun.* **313**, 85–89 (2014).

[2] Marpaung, D. *et al.* Low-power, chip-based stimulated Brillouin scattering microwave photonic filter with ultrahigh selectivity. *Optica* **2**, 76–83 (2015).

[3] Casas-Bedoya, A., Morrison, B., Pagani, M., Marpaung, D. & Eggleton, B. J. Tunable narrowband microwave photonic filter created by stimulated Brillouin scattering from a silicon nanowire. *Opt. Lett.* **40**, 4154–4157 (2015).

[4] Rasras, M. S. *et al.* Demonstration of a tunable microwave-photonic notch filter using low-loss silicon ring resonators. *J. Light. Technol.* **27**, 2105–2110 (2009).

[5] Alipour, P. *et al.* Fully reconfigurable compact RF photonic filters using high-Q silicon microdisk resonators. *Opt. Express* **19**, 15899–15907 (2011).

[6] Marpaung, D. *et al.* Si_3N_4 ring resonator-based microwave photonic notch filter with an ultrahigh peak rejection. *Opt. Express* **21**, 23286–23294 (2013).

[7] Guan, B. *et al.* CMOS compatible reconfigurable silicon photonic lattice filters using cascaded unit cells for RF-photonic processing. *IEEE J. Sel. Top. Quantum Electron.* **20**, 359–368 (2013).

[8] Dong, J. *et al.* Compact notch microwave photonic filters using on-chip integrated microring resonators. *IEEE Photonics J.* **5**, 5500307–5500307 (2013).

[9] Burla, M., Crockett, B., Chrostowski, L. & Azaña, J. Ultra-high Q multimode waveguide ring resonators for microwave photonics signal processing. In *2015 International Topical Meeting on Microwave Photonics (MWP)*, 1–4 (IEEE, 2015).

[10] Zhuang, L., Roeloffzen, C. G., Hoekman, M., Boller, K.-J. & Lowery, A. J. Programmable photonic signal processor chip for radiofrequency applications. *Optica* **2**, 854–859 (2015).

[11] Long, Y. & Wang, J. Ultra-high peak rejection notch microwave photonic filter using a single silicon microring resonator. *Opt. Express* **23**, 17739–17750 (2015).

[12] Zhuang, L. Flexible RF filter using a nonuniform SCISSOR. *Opt. Lett.* **41**, 1118–1121 (2016).

[13] Chew, S. X. *et al.* Silicon-on-insulator dual-ring notch filter for optical sideband suppression and spectral characterization. *J. Light. Technol.* **34**, 4705–4714 (2016).

[14] Liu, Y., Hotten, J., Choudhary, A., Eggleton, B. J. & Marpaung, D. All-optimized integrated RF photonic notch filter. *Opt. Lett.* **42**, 4631–4634 (2017).

[15] Fandiño, J. S., Muñoz, P., Doménech, D. & Capmany, J. A monolithic integrated photonic microwave filter. *Nat. Photonics* **11**, 124 (2017).

[16] Zheng, P. *et al.* Performances of microwave photonic notch filter based on microring resonator with dual-drive modulator. *IEEE Photonics J.* **11**, 1–13 (2018).

[17] Liu, X. *et al.* Silicon-on-insulator-based microwave photonic filter with narrowband and ultrahigh peak rejection. *Opt. Lett.* **43**, 1359–1362 (2018).

[18] Daulay, O., Liu, G. & Marpaung, D. Microwave photonic notch filter with integrated phase-to-intensity modulation transformation and optical carrier suppression. *Opt. Lett.* **46**, 488–491 (2021).

[19] Xu, X. *et al.* Advanced RF and microwave functions based on an integrated optical frequency comb source. *Opt. Express* **26**, 2569–2583 (2018).

[20] Sancho, J. *et al.* Integrable microwave filter based on a photonic crystal delay line. *Nat. Commun.* **3**, 1–9 (2012).

[21] Liu, Y., Marpaung, D., Choudhary, A. & Eggleton, B. J. Lossless and high-resolution RF photonic notch filter. *Opt. Lett.* **41**, 5306–5309 (2016).

[22] Liu, Y. *et al.* Integration of Brillouin and passive circuits for enhanced radio-frequency photonic filtering. *APL Photonics* **4**, 106103 (2019).

[23] Urick, V. J., Williams, K. J. & McKinney, J. D. *Fundamentals of microwave photonics*, vol. 1 (John Wiley & Sons, 2015).

[24] Gertler, S., Kittlaus, E. A., Otterstrom, N. T., Kharel, P. & Rakich, P. T. Microwave filtering using forward Brillouin scattering in photonic-phononic emit-receive devices. *J. Light. Technol.* **38**, 5248–5261 (2020).

[25] Gertler, S., Kharel, P., Kittlaus, E. A., Otterstrom, N. T. & Rakich, P. T. Shaping nonlinear optical response using nonlocal forward Brillouin interactions. *New Journal of Physics* **22**, 043017 (2020).

[26] Gertler, S., Kittlaus, E. A., Otterstrom, N. T. & Rakich, P. T. Tunable microwave-photonic filtering with high out-of-band rejection in silicon. *APL Photonics* **5**, 096103 (2020).

[27] Xie, Y. *et al.* System-level performance of chip-based Brillouin microwave photonic bandpass filters. *J. Light. Technol.* (2019).

[28] Kittlaus, E. A., Kharel, P., Otterstrom, N. T., Wang, Z. & Rakich, P. T. RF-photonic filters via on-chip photonic–phononic emit–receive operations. *J. Light. Technol.* **36**, 2803–2809 (2018).
